# Paxillin mediates ATP-induced activation of P2X7 receptor and NLRP3 inflammasome

**DOI:** 10.1186/s12915-020-00918-w

**Published:** 2020-11-26

**Authors:** Wenbiao Wang, Dingwen Hu, Yuqian Feng, Caifeng Wu, Yunting Song, Weiyong Liu, Aixin Li, Yingchong Wang, Keli Chen, Mingfu Tian, Feng Xiao, Qi Zhang, Weijie Chen, Pan Pan, Pin Wan, Yingle Liu, Huiyao Lan, Kailang Wu, Jianguo Wu

**Affiliations:** 1 Guangdong Provincial Key Laboratory of Virology, Institute of Medical Microbiology, Jinan University, Guangzhou, 510632 China; 2 State Key Laboratory of Virology, College of Life Sciences, Wuhan University, Wuhan, 430072 China; 3 Department of Medicine & Therapeutics, Li Ka Shing Institute of Health Sciences, Lui Che Woo Institute of Innovative Medicine, The Chinese University of Hong Kong, Sha Tin, Hong Kong, China; 4 Department of Clinical Laboratory, Tongji Hospital, Tongji Medical College, Huazhong University of Science and Technology, Wuhan, 430030 China

**Keywords:** Adenosine triphosphate, ATP, P2X7 receptor, Paxillin, The NACHT, LRR, and PYD domain-containing protein 3, NLRP3, Ubiquitin-specific peptidase 13, USP13

## Abstract

**Background:**

Extracellular adenosine triphosphate (ATP), a key danger-associated molecular pattern (DAMP) molecule, is released to the extracellular medium during inflammation by injured parenchymal cells, dying leukocytes, and activated platelets. ATP directly activates the plasma membrane channel P2X7 receptor (P2X7R), leading to an intracellular influx of K^+^, a key trigger inducing NLRP3 inflammasome activation. However, the mechanism underlying P2X7R-mediated activation of NLRP3 inflammasome is poorly understood, and additional molecular mediators have not been identified. Here, we demonstrate that Paxillin is the molecule connecting the P2X7 receptor and NLRP3 inflammasome through protein interactions.

**Results:**

We show a distinct mechanism by which Paxillin promotes ATP-induced activation of the P2X7 receptor and NLRP3 inflammasome. Extracellular ATP induces Paxillin phosphorylation and then facilitates Paxillin-NLRP3 interaction. Interestingly, Paxillin enhances NLRP3 deubiquitination and activates NLRP3 inflammasome upon ATP treatment and K^+^ efflux. Moreover, we demonstrated that USP13 is a key enzyme for Paxillin-mediated NLRP3 deubiquitination upon ATP treatment. Notably, extracellular ATP promotes Paxillin and NLRP3 migration from the cytosol to the plasma membrane and facilitates P2X7R-Paxillin interaction and PaxillinNLRP3 association, resulting in the formation of the P2X7R-Paxillin-NLRP3 complex. Functionally, Paxillin is essential for ATP-induced NLRP3 inflammasome activation in mouse BMDMs and BMDCs as well as in human PBMCs and THP-1-differentiated macrophages.

**Conclusions:**

We have identified paxillin as a mediator of NLRP3 inflammasome activation. Paxillin plays key roles in ATP-induced activation of the P2X7 receptor and NLRP3 inflammasome by facilitating the formation of the P2X7R-Paxillin-NLRP3 complex.

**Supplementary information:**

The online version contains supplementary material available at 10.1186/s12915-020-00918-w.

## Background

The NACHT, LRR, and PYD domain-containing protein 3 (NLRP3), one of the host pattern recognition receptors (PRRs), recognizes pathogen-associated molecular patterns (PAMPs) and danger-associated molecular patterns (DAMPs). NLRP3 controls maturation and secretion of interleukin-1β (IL-1β) and IL-18, two pleiotropic cytokines playing crucial roles in innate immune and inflammatory responses as well as instructing adaptive immune responses [1]. NLRP3 (the cytoplasmic sensor molecular) together with apoptosis-associated speck-like protein with the CARD domain (ASC) (the adaptor protein) promote the cleavage of the pro-Caspase-1 (the effector protein) to generate active subunits p20 and p10, which regulate the maturation of IL-1β [2]. NLRP3 requires two signals for canonical activation and for IL-1β secretion: the first signal primes the cell to express NLRP3 and pro-IL-1β mRNAs, and the second signal induces inflammasome assembly and activation [3]. NLRP3 forms a scaffold with ASC to provide a molecular platform for the activation of pro-Caspase-1, which collectively comprises the inflammasome [4]. Activated Caspase-1 cleaves pro-IL-1β into active IL-1β, which is then secreted.

Extracellular adenosine triphosphate (ATP), a key DAMP, is released during inflammation by injured parenchymal cells, dying leukocytes, and activated platelets [5]. Activation of NLRP3 inflammasome requires the activation of the P2X7 receptor (P2X7R), a plasma membrane channel that is directly activated by extracellular ATP [6, 7]. After binding with ATP, the channel opens and induces transmembrane ion fluxes of K^+^, which is a key trigger of the NLRP3 inflammasome activation [8, 9]. However, the mechanism underlying this regulation is not fully understood and the molecule connecting the P2X7 receptor and NLRP3 inflammasome is not identified.

Paxillin is a multi-domain protein that localizes to the extracellular matrix (ECM) and plays important roles in cell motility, adhesion, migration, and growth control [10]. The primary function of Paxillin is as a molecular adapter or scaffold protein that provides multiple docking sites at the plasma membrane for many proteins, such as focal adhesion kinase (FAK) [11] and extracellular signal-regulated kinase (ERK) [12]. It is also involved in the efficient processing of Integrin- and growth factor-mediated signals derived from the extracellular environment [13].

This study demonstrates a distinct mechanism underlying ATP-induced activation of the P2X7 receptor and NLRP3 inflammasome mediated by Paxillin. Extracellular ATP induces Paxillin phosphorylation, facilitates Paxillin-NLRP3 interaction, and promotes NLRP3 deubiquitination, thereby activating the NLRP3 inflammasome. Additionally, ATP promotes Paxillin and NLRP3 membrane migration and facilitates P2X7R-Paxillin interaction, resulting in the formation of the P2X7R-Paxillin-NLRP3 complex. Moreover, ubiquitin-specific peptidase 13 (USP13) is a key enzyme for Paxillin-mediated NLRP3 deubiquitination [14]. Paxillin functionally is essential for ATP- and Nigericin-induced NLRP3 inflammasome activation. Taken together, these results demonstrate that Paxillin promotes ATP-induced activation of the P2X7 receptor and NLRP3 inflammasome by facilitating the formation of the P2X7R-Paxillin-NLRP3 complex.

## Results

### Paxillin binds directly to the LRR domain of NLRP3 in the cytosol

**Fig. 1:**
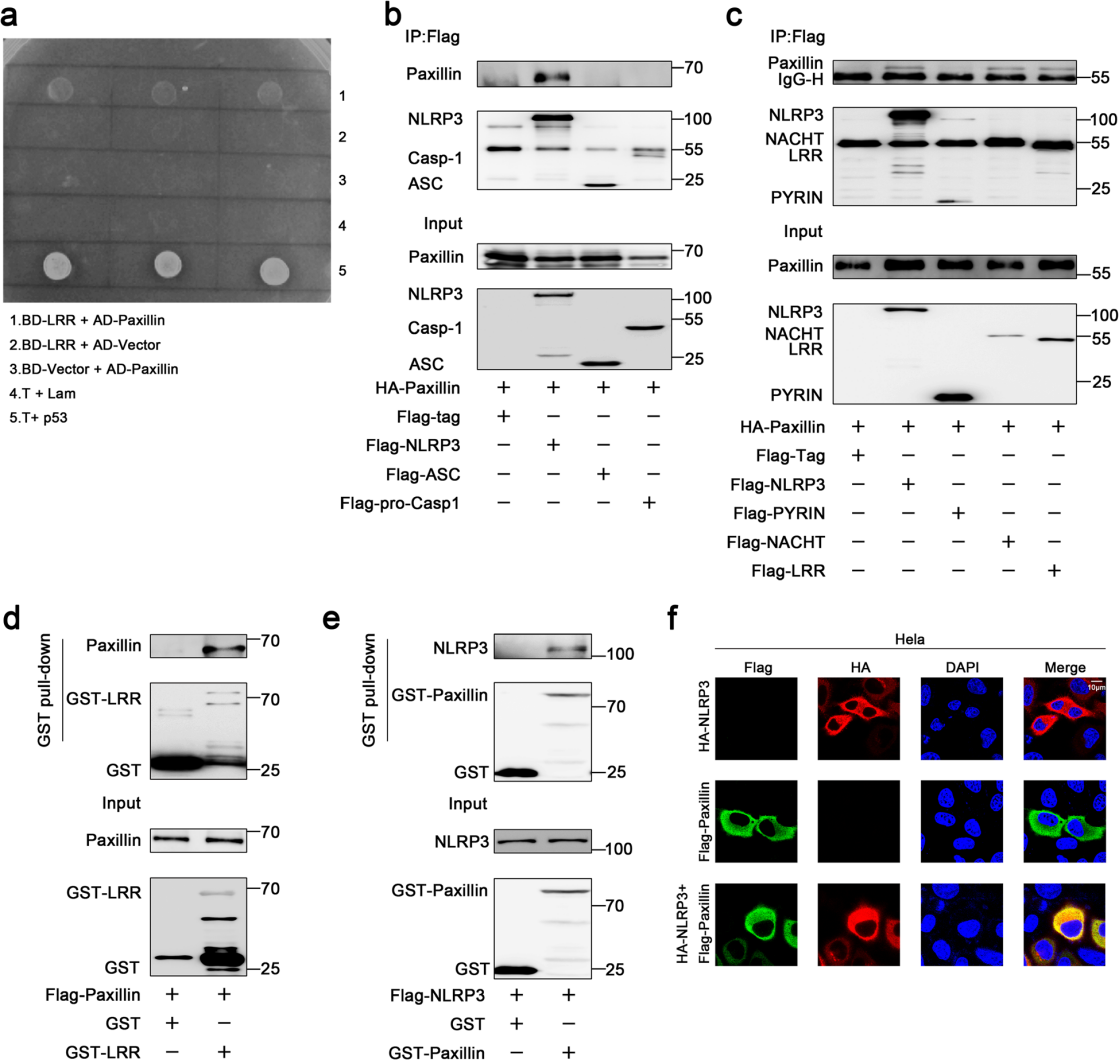
Paxillin binds directly to the LRR domain of NLRP3 in the cytosol. **a** Identification of NLRP3 LRR domain-Paxillin interaction by yeast two-hybrid analysis. Yeast strain AH109 cells were transformed with the combination of BD and AD plasmid, as indicated (1–3). Transformed yeast cells were first grown on the SD-minus Trp/Leu plates for 3 days. The colony of yeast was then streaked on SD-minus Trp/Leu/Ade/His plates (QDO). BD-p53 and AD-T (5) was used as a positive control and BD-Lam and AD-T (4) as a negative control. **b** HEK293T cells were co-transfected with pHA-Paxillin and pFlag-Vector, pFlag-NLRP3, pFlag-ASC, and pFlag-Casp-1. Lysates were prepared and subjected to IP using an anti-Flag antibody and analyzed by immunoblotting using an anti-HA or anti-Flag antibody (top) or subjected directly to Western blot using an anti-Flag or anti-HA antibody (as input) (bottom). **c** HEK293T cells were co-transfected with pHA-Paxillin and pFlag-Vector, pFlag-NLRP3, pFlag-PYRIN, pFlag-NACHT, and pFlag-LRR. Lysates were prepared and subjected to IP using an anti-Flag antibody and analyzed by immunoblotting using an anti-HA or anti-Flag antibody (top) or subjected directly to Western blot using an anti-Flag or anti-HA antibody (as input) (bottom). **d** Extracts from HEK293T cells transfected with Flag-Paxillin were incubated with 10 μg GST proteins or GST-LRR protein that was incubated with glutathione-Sepharose beads. The mixture was washed three times and then analyzed by immunoblotting using anti-Flag and anti-GST antibody (top). Lysates from transfected HEK293T cells were analyzed by immunoblotting using anti-Flag antibody (as input) (bottom). **e** Extracts from HEK293T cells transfected with Flag-NLRP3 were incubated with 10 μg GST proteins or GST-Paxillin protein which was incubated with glutathione-Sepharose beads. The mixture was washed three times and then analyzed by immunoblotting using anti-Flag and anti-GST antibody (top). Lysates from transfected HEK293T cells were analyzed by immunoblotting using anti-Flag antibody (as input) (bottom). **f** HeLa cells were transfected with pFlag-Paxillin and pHA-NLRP3 or co-transfected with pFlag-Paxillin and pHA-NLRP3. Subcellular localization of Flag-Paxillin (green), HA-NLRP3 (red), and the nucleus marker DAPI (blue) was examined by confocal microscopy

We have recently revealed that several proteins control the NLRP3 inflammasome activation through different mechanisms [15–18]. Here, we further showed that Paxillin interacted with NLRP3 based on a yeast two-hybrid screen (Fig. 1a). Co-immunoprecipitation (Co-IP) assays showed that Paxillin interacted with NLRP3, but not with ASC or pro-Caspase-1 (Fig. 1b). NLRP3 contains several prototypic domains, including a PYRIN domain, an NACHT domain, and seven LRR domains [19]. Paxillin was co-precipitated with NLRP3, NACHT, and LRR, but not with PYRIN (Fig. 1c). Glutathione S-transferase (GST) pull-down assays showed that GST-LRR was pulled down with Paxillin (Fig. 1d) and GST-Paxillin was pulled down with NLRP3 (Fig. 1e), suggesting that Paxillin directly binds to the NLRP3 LRR domain. Confocal microscopy showed that NLRP3 alone or Paxillin alone was diffusely distributed in the cytosol, whereas NLRP3 and Paxillin together were co-localized in the cytosol (Fig. 1f). Collectively, these data demonstrate that Paxillin binds directly to the LRR domain of NLRP3 in the cytosol.

### ATP promotes Paxillin phosphorylation and Paxillin-NLRP3 interaction

**Fig. 2:**
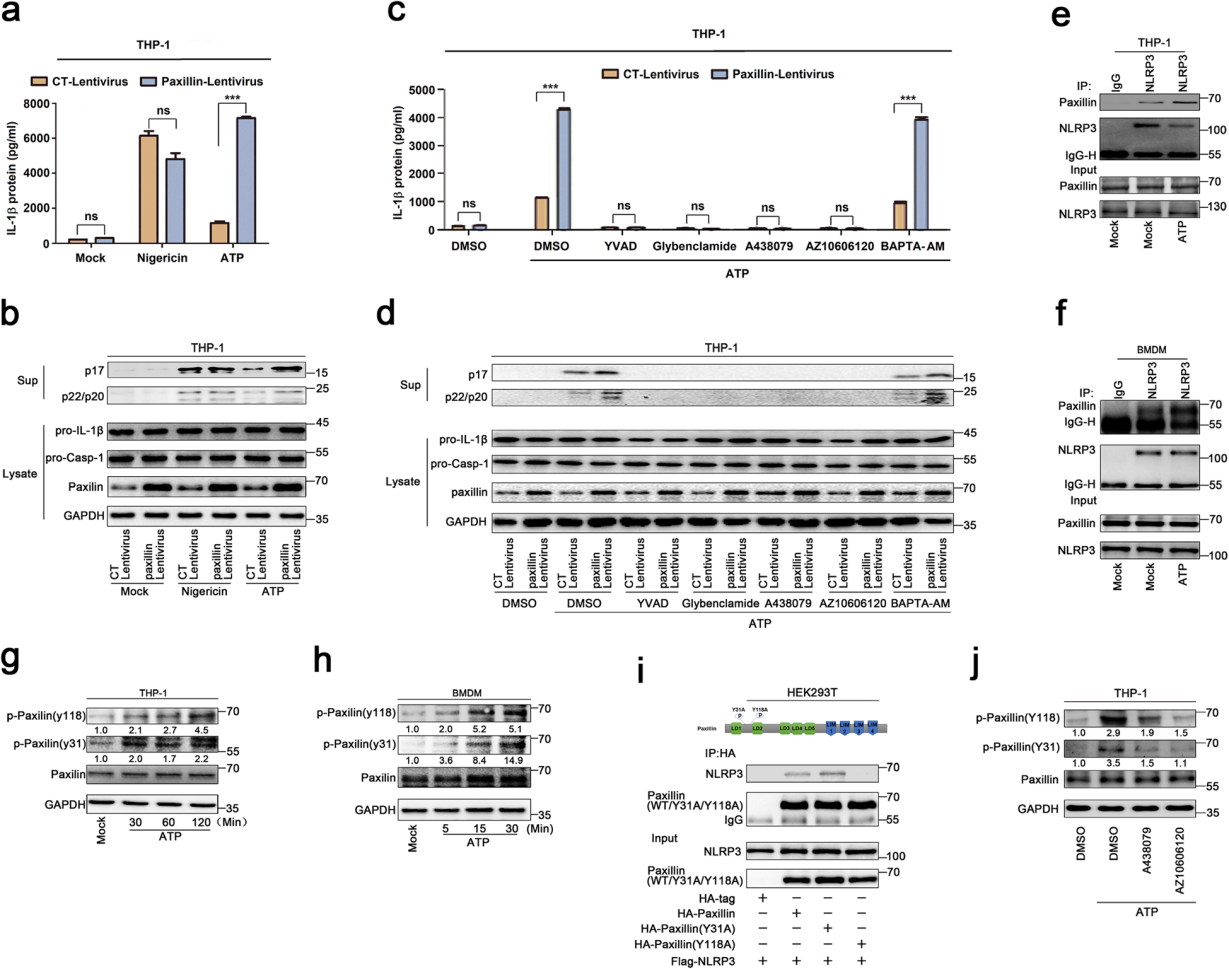
ATP promotes Paxillin phosphorylation and Paxillin-NLRP3 interaction. **a**–**d** THP-1 cells stably expressing control lentivirus or Paxillin lentivirus were generated and differentiated into macrophages, which were then treated with ATP (5 mM) or Nigericin (2 μM) for 2 h (**a**, **b**) and treated with DMSO, YVAD (50 μM), Glybenclamide (25 μg/ml), A438079 (10 μM), AZ10606120 (10 μM), and BAPTA-AM (30 μM) for 1 h before the treatment with ATP (5 mM) for 2 h (**c**, **d**). IL-1β levels in supernatants were determined by ELISA (**a**, **c**). Mature IL-1β (p17) and cleaved Casp-1 (p22/p20) in supernatants or Paxillin, pro-IL-1β, and pro-Casp-1 in lysates were determined by Western blot (**b**, **d**). **e** TPA-differentiated THP-1 macrophages were treated with DMSO or ATP (5 mM) for 2 h. Lysates were prepared and subjected to IP (top) or subjected to Western blot (as input) (bottom). **f** BMDM cells prepared from C57BL/6 mice bone marrow were treated with LPS (1 μg/ml) for 6 h, and the primed BMDMs were stimulated by DMSO or ATP (5 mM) for 30 min. Lysates were prepared and subjected to IP (top) or subjected to Western blot (as input) (bottom). **g**, **h** TPA-differentiated THP-1 macrophages were treated with ATP (5 mM) for 30, 60, and 120 min (**g**). LPS-primed BMDMs were stimulated by ATP (5 mM) for 5, 15, and 30 min (**h**). The protein level of p-Paxillin(Y118), p-Paxillin(Y31), Paxillin, and GAPDH was determined by Western blot. **i** HEK293T cells were co-transfected with pFlag-NLRP3 and pHA-Vector, pHA-Paxillin, pHA-Paxillin-(Y31A), and pHA-Paxillin-(Y118A). Lysates were prepared and subjected to IP (top) or subjected to Western blot (as input) (bottom). **j** TPA-differentiated THP-1 macrophages were treated with DMSO, A438079 (10 μM), and AZ10606120 (10 μM) for 1 h before the treatment with ATP (5 mM) for 2 h. The protein level of p-Paxillin(Y118), p-Paxillin (Y31), Paxillin, and GAPDH was determined by Western blot. Data shown are means ± SEMs; ***p < 0.0001. ns, no significance. Densitometry of the blots was measured by ImageJ

ATP and Nigericin are common activators of the NLRP3 inflammasome [20]. Human acute monocytic leukemia cells (THP-1) that stably expressed Paxillin were generated by infection with Paxillin-Lentivirus, which were then treated with ATP or Nigericin. ATP-induced inflammasome activation, as indicated by IL-1β secretion, IL-1β maturation, and Caspase-1 cleavage, was further promoted by Paxillin, whereas Nigericin-induced inflammasome activation was not affected by Paxillin (Fig. 2a, b), suggesting that Paxillin specifically promotes ATP-induced NLRP3 inflammasome activation. As ATP stimulates P2X7R ATP-gated ion channel and triggers K^+^ efflux, leading to inflammasome activation [8], we evaluated the effect of Paxillin on NLRP3 inflammasome activation. In Paxillin stable THP-1 cells, IL-1β secretion, IL-1β maturation, and Caspase-1 cleavage were induced by ATP and further facilitated by Paxillin in the presence of dimethylsulphoxide (DMSO) or BAPTA-AM (Ca^2+^ chelator), whereas activations of IL-1β and Caspase-1 were not induced by ATP or Paxillin in the presence of YVAD (Caspase-1 inhibitor), Glybenclamide (K^+^ efflux inhibitor), A438079 (P2X7R antagonist), or AZ10606120 (P2X7R-negative allosteric modulator) (Fig. 2c, d). These results indicate that like ATP, Paxillin promotes IL-1β and Caspase-1 activation depending on P2X7R, K^+^ efflux, and Casp-1, and suggest that Paxillin facilitates ATP-induced NLRP3 inflammasome activation.

The mechanism by which Paxillin regulates ATP-induced NLRP3 inflammasome was investigated. As Paxillin interacts with NLRP3, we explored whether ATP affects this interaction. Paxillin-NLRP3 interaction was enhanced by ATP in TPA-differentiated THP-1 macrophages (Fig. 2e) and LPS-primed mouse bone marrow-derived macrophages (BMDMs) (Fig. 2f). Paxillin is a phosphorylated molecule and plays an important role in cell adhesion and migration [21], and phosphorylations of Paxillin at Y31 and Y118 were essential for the generation of protein binding module [11]. We demonstrated that Paxillin Y31 and Y118 phosphorylations were promoted by ATP in THP-1 differentiated macrophages (Fig 2g) and LPS-primed BMDMs (Fig. 2h). Like Paxillin, Paxillin(Y31A) interacted with NLRP3, whereas Paxillin (Y118A) failed to interact with NLRP3 (Fig. 2i), suggesting an essential role of Y118 in Paxillin-NLRP3 interaction. Moreover, ATP-induced Paxillin Y118 and Y31 phosphorylation were attenuated by P2X7R antagonists (A438079) and P2X7R-negative allosteric modulator (AZ10606120) (Fig. 2j), indicating that P2X7R is involved in Paxillin Y118 phosphorylation. Therefore, ATP- and P2X7R-induced Paxillin Y118 phosphorylation is essential for Paxillin-NLRP3 interaction.

### ATP stimulates P2X7R-Paxillin interaction

**Fig. 3:**
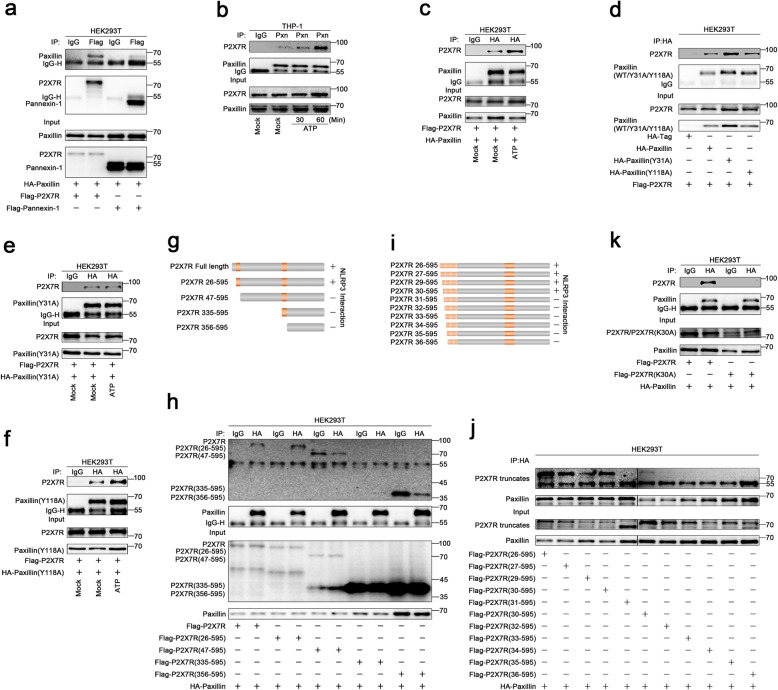
ATP stimulates P2X7R-Paxillin interaction. **a** HEK293T cells were co-transfected with pFlag-P2X7R and pHA-Paxillin or pFlag-Pannexin-1 and pHA-Paxillin. **b** TPA-differentiated THP-1 macrophages were mock-treated or treated with ATP (5 mM) for 30 and 60 min. **c** HEK293T cells were co-transfected with pFlag-P2X7R and pHA-Paxillin and treated with DMSO or ATP (5 mM) for 2 h. **d** HEK293T cells were co-transfected with pFlag-P2X7R and pHA-Vector, pHA-Paxillin, pHA-Paxillin(Y31A), and pHA-Paxillin(Y118A). **e** HEK293T cells were co-transfected with pFlag-P2X7R and pHA-paxillin(Y31A) and treated with DMSO or ATP (5 mM) for 2 h. **f** HEK293T cells were co-transfected with pFlag-P2X7R and pHA-Paxillin(Y118A) and treated with DMSO or ATP (5 mM) for 2 h. **g** The diagrams of P2X7R, P2X7R(26–595), P2X7R(47–595), P2X7R(335–595), and P2X7R(356–595) (left). **h** HEK293T cells were co-transfected with pHA-Paxillin and pFlag-P2X7R, pFlag-P2X7R(26–595), pFlag-P2X7R(47–595), pFlag-P2X7R(335–595), and pFlag-P2X7R(356–595). **i** The diagrams of P2X7R(26/27/29/30/31/32/33/34/35/36–595). **j** HEK293T cells were co-transfected with 2 μg pHA-Paxillin and 6 μg pFlag-P2X7R(26–595), pFlag-P2X7R(27–595), pFlag-P2X7R(29–595), pFlag-P2X7R(30–595), pFlag-P2X7R(31–595), pFlag-P2X7R(30–595), pFlag-P2X7R(32–595), pFlag-P2X7R(33–595), pFlag-P2X7R(34–595), pFlag-P2X7R(35–595), and pFlag-P2X7R(36–595). **k** HEK293T cells were co-transfected with pFlag-P2X7R and pHA-Paxillin or pFlag-P2X7R(K30A) and pHA-Paxillin

Extracellular ATP stimulates P2X7R ATP-gated ion channel and inducing gradual recruitment of Pannexin-1 membrane pore [22]. As the downstream adaptor of Integrin, Paxillin functions as a scaffold for the recruitment of proteins into a complex [9], we thus explored whether Paxillin interacts with P2X7R. The result showed that Paxillin interacted with P2X7R and Pannexin-1 (Fig. 3a), and P2X7R-Paxillin interaction was promoted by ATP (Fig. 3b, c). Like Paxillin, Paxillin (Y31A) and Paxillin (Y118A) interacted with P2X7R (Fig. 3d). Notably, P2X7R-Paxillin (Y31A) interaction was not facilitated by ATP (Fig. 3e), whereas P2X7R-Paxillin (Y118A) interaction was promoted by ATP (Fig. 3f). These results demonstrate that phosphorylation site Y31 is involved in ATP-induced P2X7R-Paxillin interaction.

P2X7 subunit contains the intracellular N-termini (1–25aa), the C-termini (357–595aa), the P2X7 receptor extracellular domain (47–334aa), and two transmembrane domains TM1(26–46aa) and TM2(335–355aa) [23]. Accordingly, five plasmids expressing P2X7R and deletion mutants were constructed (Fig. 3g). Paxillin interacted with P2X7R and P2X7R(26–595), whereas Paxillin failed to interact with P2X7R(47–595), P2X7R(335–595), or P2X7R(356–595), suggesting that Paxillin interacts with 26aa–46aa of P2X7R (Fig. 3h). To narrow down the interaction region, ten plasmids expressing truncated P2X7R proteins were constructed (Fig. 3i). Paxillin interacted with P2X7R(26–595), P2X7R(27–595), P2X7R(29–595), and P2X7R(30–595), but it failed to associate with P2X7R(31–595), P2X7R(32–595), P2X7R(33–595), P2X7R(34–595), P2X7R(35–595), or P2X7R(36–595) (Fig. 3j), indicating that P2X7R K30 is required for Paxillin interaction. To confirm this result, K30 was mutated to A30 to generate mutant P2X7R(K30A). Notably, unlike P2X7R, P2X7R(K30A) failed to interact with Paxillin (Fig. 3k), confirming that K30 is essential for the interaction with Paxillin. Therefore, ATP stimulates P2X7R-Paxillin interaction and promotes Paxillin-NLRP3 interaction, and thus, Paxillin plays an essential role in P2X7R-Paxillin-NLRP3 complex assembly upon ATP induction.

### Paxillin promotes NLRP3 deubiquitination depending on extracellular ATP and K^+^ efflux

**Fig. 4:**
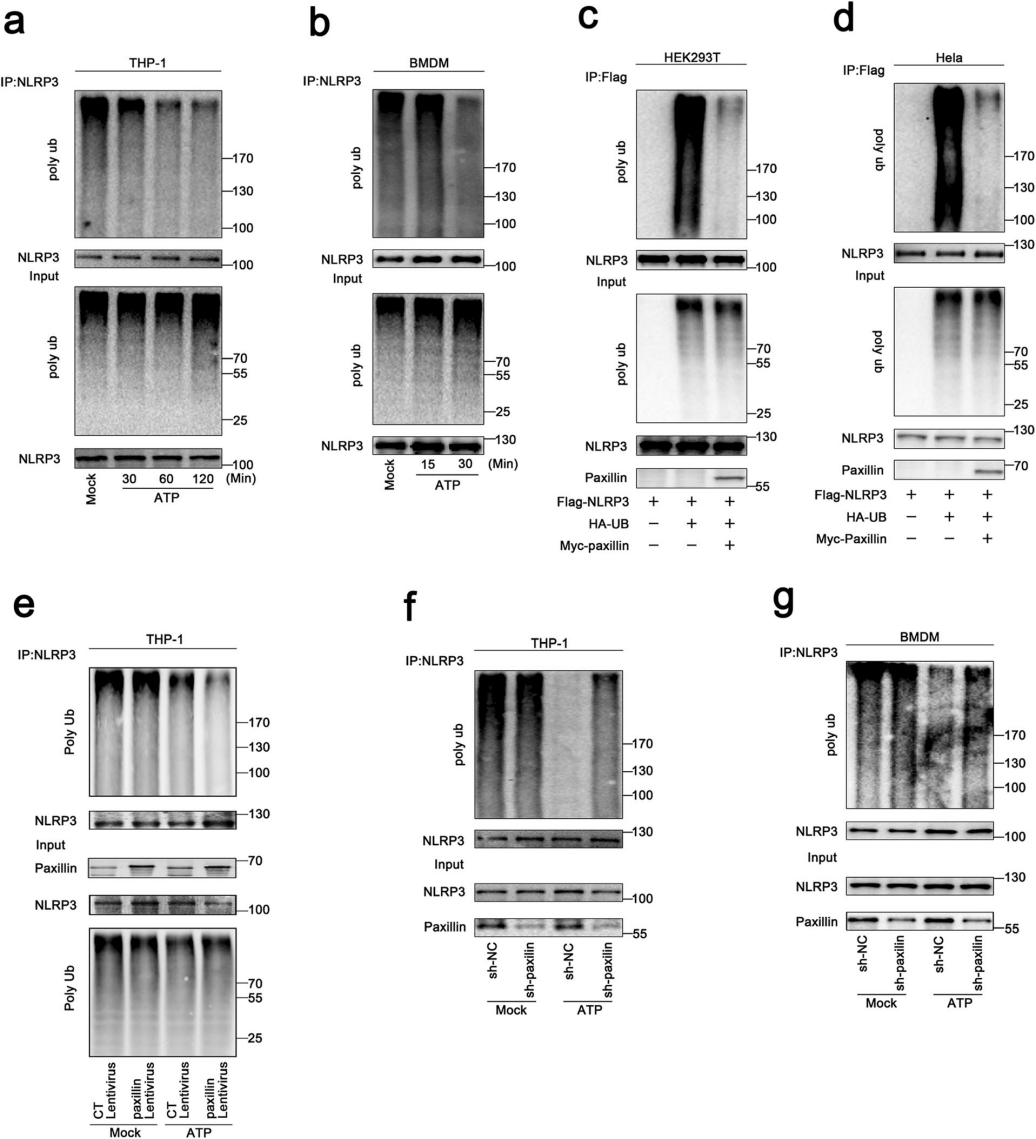
Paxillin promotes NLRP3 deubiquitination depending on extracellular ATP. **a** TPA-differentiated THP-1 macrophages were treated with ATP (5 mM) for 30, 60, and 120 min. **b** BMDM cells prepared from C57BL/6 mice bone marrow were treated with LPS (1 μg/ml) for 6 h, and the primed BMDM cells were stimulated by ATP (5 mM) for 15 and 30 min. **c**, **d** HEK293T cells (**c**) and Hela cells (**d**) were co-transfected with pFlag-NLRP3, pHA-Ubiquitin, or pMyc-Paxillin. **e** THP-1 cells stably expressing control lentivirus or Paxillin lentivirus were generated and differentiated into macrophages, which were then treated with ATP (5 mM) for 2 h. **f** THP-1 cells stably expressing shRNA targeting Paxillin were generated and differentiated into macrophages, which were then treated with ATP (5 mM) for 2 h. **g** BMDMs prepared from C57BL/6 mice bone marrow were infected by lentivirus that express shRNA targeting Paxillin for 3 days. Before stimulation, the BMDMs were treated with LPS (1 μg/ml) for 6 h, and the primed BMDMs were stimulated by ATP (5 mM) for 30 min

As NLRP3 deubiquitination is important for the inflammasome activation [24], here, the roles of ATP and Paxillin in NLRP3 deubiquitination were evaluated. The level of NLRP3 ubiquitination was attenuated by ATP in THP-1-differentiated macrophages and LPS-primed BMDMs (Fig. 4a, b). Similarly, the abundance of NLRP3 ubiquitination was reduced by Paxillin in HEK293T cells and HeLa cells (Fig. 4c, d). Human acute monocytic leukemia cells (THP-1) stably expressed Paxillin were generated by infection with Paxillin-Lentivirus, which were then treated with ATP. The results showed that Paxillin could not affect NLRP3 deubiquitination in the absence of ATP, whereas it promoted ATP-induced NLRP3 deubiquitination in THP-1 cells (Fig. 4e). In addition, human acute monocytic leukemia cells (THP-1) stably expressed the short hairpin RNAs (shRNAs) against Paxillin (sh-Paxillin) were generated by infection with sh-Paxillin-Lentivirus and the short hairpin RNA (shRNA) against GFP (sh-NC) were generated by infection with sh-NC-Lentivirus. ATP-induced NLRP3 deubiquitination was detected in the absence of sh-Paxillin, whereas it was repressed by sh-Paxillin in THP-1 cells and BMDMs (Fig. 4f, g). Collectively, these results demonstrate that Paxillin promotes ATP-induced NLRP3 deubiquitination.

**Fig. 5:**
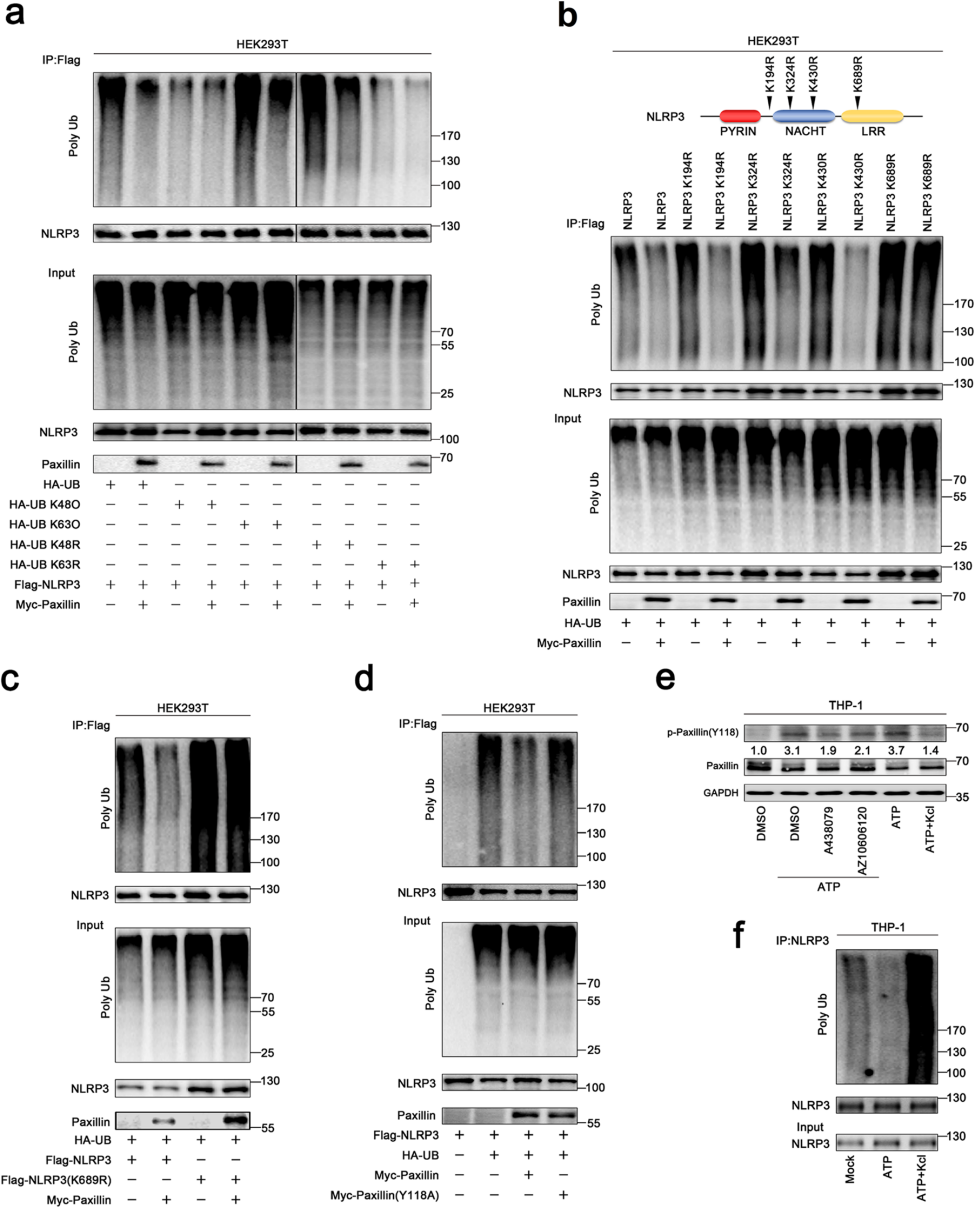
Paxillin promotes NLRP3 K63-linked deubiquitination depending on extracellular ATP and K^+^ efflux. **a** HEK293T cells were co-transfected with pFlag-NLRP3 and pHA-UB, pHA-UB(K48O), pHA-UB(K63O), pHA-UB(K48R), pHA-UB(K63R), or pMyc-Paxillin. **b** HEK293T cells were co-transfected with pHA-UB and pFlag-NLRP3, pFlag-NLRP3(K194R), pFlag-NLRP3(K324R), pFlag-NLRP3(K403R), pFlag-NLRP3(K689R), or pMyc-Paxillin. **c** HEK293T cells were co-transfected with pHA-UB and pFlag-NLRP3, pFlag-NLRP3(K689R), or pMyc-Paxillin. **d** HEK293T cells were co-transfected with pFlag-NLRP3 and pHA-UB, pMyc-Paxillin, or pMyc-Paxillin(Y118A). **e** TPA-differentiated THP-1 macrophages were treated with DMSO, A438079 (10 μM), or AZ10606120 (10 μM) for 1 h before the treatment with ATP (5 mM) for 2 h or treated with ATP (5 mM) for 2 h and with/without 50 mM extracellular KCl. The protein levels of p-Paxillin-(y118), Paxillin, and GAPDH were determined by Western blotting. **f** TPA-differentiated THP-1 macrophages were treated with ATP (5 mM) for 2 h in the presence or absence of 50 mM extracellular KCl. Lysates were prepared and subjected to denature-IP (top) or subjected to Western blot (as input) (bottom) (a–m). Densitometry of the blots was measured by ImageJ

To determine the nature of NLRP3 ubiquitination, two ubiquitin mutants that retain only a single lysine residue (KO) and two ubiquitin mutants in which only one lysine residue is mutated (KR) were generated. NLRP3 was ubiquitinated by UB, UB(K63O), and UB(K48R), whereas it failed to be ubiquitinated by UB(K48O) or UB(K63R), and NLRP3 K63-linked ubiquitination was attenuated by Paxillin (Fig. 5a), indicating that NLRP3 ubiquitination is K63-linked and Paxillin facilitates the removal of NLRP3 K63-linked ubiquitination.

Moreover, the site of NLPR3 ubiquitination was determined by constructing and analyzing four only one lysine residual mutants (KR) of NLPR3 (Fig. 5b, top). Ubiquitinations of NLRP3, NLRP3(K194R), and NLRP3(K430R) were attenuated by Paxillin; ubiquitination of NLRP3(K324R) was downregulated by Paxillin, whereas ubiquitination of NLRP3(K689R) was not affected by Paxillin (Fig. 5b, bottom), and Paxillin attenuated NLRP3 ubiquitination but failed to reduce NLRP3(K689R) ubiquitination (Fig. 5c), showing that Paxillin specifically removes NLRP3 ubiquitination at K689. As Paxillin Y118 phosphorylation is required for Paxillin-NLRP3 interaction, we evaluated its effect on NLRP3 deubiquitination. NLRP3 ubiquitination was notably attenuated by Paxillin, whereas it was not affected by Paxillin(Y118A) (Fig. 5d), indicating that Paxillin Y118 phosphorylation is required for NLRP3 deubiquitination.

As ATP-induced Paxillin Y118 phosphorylation is essential for Paxillin-NLRP3 interaction and deubiquitination, we further explored the effect of K^+^ efflux on Paxillin Y118 phosphorylation. Paxillin Y118 phosphorylation was induced by ATP, but such induction was attenuated by P2X7R antagonists (A438079), P2X7R-negative allosteric modulator (AZ10606120), and KCl treatment (Fig. 5e). Furthermore, the role of K^+^ efflux in NLRP3 deubiquitination was revealed. Notably, ATP attenuated NLRP3 ubiquitination, but such attenuation was suppressed by KCl (Fig. 5f), demonstrating that K^+^ efflux is important for Paxillin- and ATP-induced deubiquitination of NLRP3. Together, we demonstrate that Paxillin facilitates the removal of NLRP3 K63-linked ubiquitination at K689 and that ATP and K^+^ efflux are critical for Paxillin-mediated NLRP3 deubiquitination.

### USP13 is essential for Paxillin-mediated NLRP3 deubiquitination upon ATP treatment

**Fig. 6:**
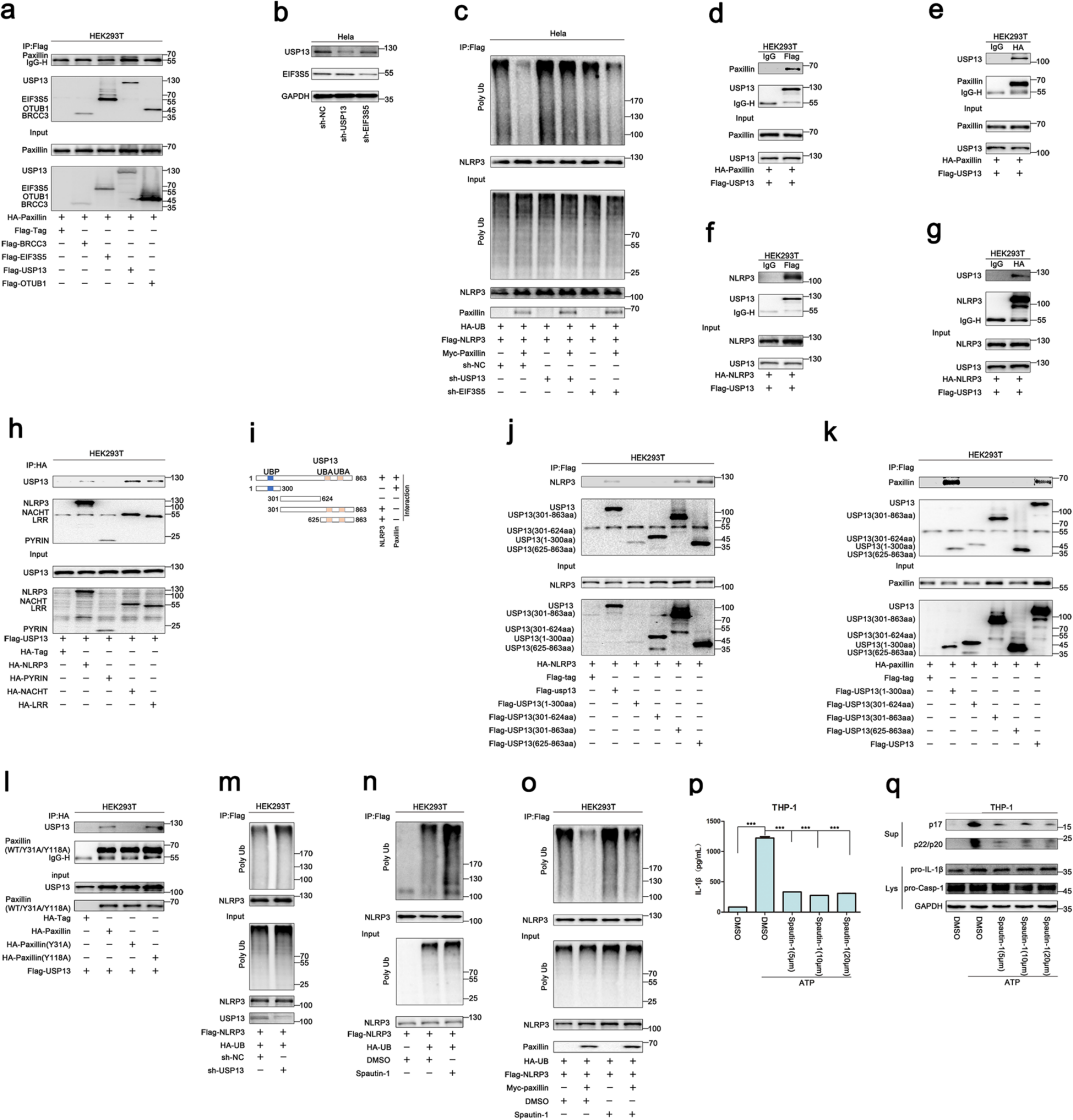
USP13 is essential for Paxillin-mediated NLRP3 deubiquitination upon ATP treatment. **a** HEK293T cells were co-transfected with pHA-Paxillin and pFlag-BRCC3, pFlag-EIF3S5, pFlag-USP13, or pFlag-OTUB1. Lysates were prepared and subjected to IP (top) or subjected to Western blot (as input) (bottom). **b** Hela cells stably expressing sh-USP13 or sh-EIF3S5 were generated and analyzed. **c** The stable Hela cells were co-transfected with pFlag-NLRP3, pHA-UB, or pMyc-Paxillin. Lysates were prepared and subjected to denature-IP (top) or subjected to Western blot (bottom). **d**, **e** HEK293T cells were transfected with pFlag-USP13 and pHA-Paxillin. **f**, **g** HEK293T cells were transfected with pFlag-USP13 and pHA-NLRP3. Lysates were prepared and subjected to IP (top) or subjected to Western blot (bottom) (**d**–**g**). **h** HEK293T cells were co-transfected with pFlag-USP13 and pHA-NLRP3 or pHA-NLRP3 mutants. **i** The diagrams of USP13 and its mutants, as indicated. **j** HEK293T cells were co-transfected with pHA-NLRP3 and pFlag-USP13 or pFlag-USP13 mutants. **k** HEK293T cells were co-transfected with pHA-Paxillin and pFlag-USP13 or pFlag-USP13 mutants. **l** HEK293T cells were co-transfected with pFlag-USP13 and pHA-Paxillin or pHA-Paxillin mutants. Lysates were prepared and subjected to IP (top) or subjected to Western blot (bottom) (**h**, **j**–**l**). **m** HEK293T cells stably expressing sh-USP13 were generated. The stable cells were co-transfected with pFlag-NLRP3 and pHA-UB. **n** The stable HEK293T cells were co-transfected with pFlag-NLRP3 or pHA-UB with or without Spautin-1 (20 μM) for 6 h. **o** The stable HEK293T cells were co-transfected with pFlag-NLRP3, pHA-UB, or pMyc-Paxillin with or without Spautin-1 (20 μM) for 6 h. Lysates were prepared and subjected to denature-IP (top) or subjected to Western blot (bottom) (**m**–**o**). **p**, **q** TPA-differentiated THP-1 macrophages were treated with ATP (5 mM) for 2 h with/without Spautin-1 (5, 10, and 20 μM). IL-1β in supernatants was determined by ELISA (**p**). Mature IL-1β(p17) and cleaved Caspase-1(p22/p20) in supernatants and Paxillin, pro-IL-1β, and pro-Casp-1 in lysates were determined by Western blot (**q**). Data shown are means ± SEMs; ***p < 0.0001

Next, the enzyme required for Paxillin-mediated NLRP3 deubiquitination was then determined. Deubiquitinating enzymes, such as eukaryotic translation initiation factor 3, subunit 5 (EIF3S5), ubiquitin-specific peptidase 13 (USP13), and OTU domain-containing ubiquitin aldehyde-binding protein 1 (OTUB1), were potentially involved in the deubiquitination of NLRP3 [24]. Here, we explored the roles of these enzymes in NLRP3 deubiquitination. Paxillin interacted with EIF3S5 and USP13 (Fig. 6a), showing that EIF3S5 and USP13 might have participated in Paxillin-mediated NLRP3 deubiquitination. To confirm the roles of EIF3S5 and USP13 in the regulation of NLRP3 activation, Hela cells stably expressing sh-RNA targeting human EIF3S5 (sh-EIF3S5) and human USP13 (sh-USP13) were generated and analyzed (Fig. 6b). NLRP3 ubiquitination was attenuated by Paxillin in the presence of sh-NC and sh-EIF3S5, whereas it was relatively unaffected by Paxillin in the presence of sh-USP13 (Fig. 6c), indicating that USP13 is required for Paxillin-mediated NLRP3 deubiquitination. Moreover, Co-IP assays showed that Paxillin and USP13 interacted with each other (Fig. 6d, e). Co-IP assays showed that NLRP3 and USP13 interacted with each other (Fig. 6f, g). Like NLRP3, the NLRP3 NACHT domain and LRR domain interacted with USP13, but the NLRP3 PYRIN domain failed to interact with USP13 (Fig. 6h).

Moreover, the domains of USP13 required for USP13-NLRP3 interaction and USP13-Paxillin association were assessed by constructing and analyzing plasmids encoding for WT USP13(1–863) and four truncated proteins (Fig. 6i). Like WT USP13(1–863), USP13(301–863) and USP13(625–863) interacted with NLRP3, but USP13(1–300) or USP13(301–624) failed to interact with NLRP3 (Fig. 6j), indicating that 625aa–863aa of USP13 are involved in USP13-NLRP3 interaction. Additionally, USP13(1–863) and USP13(1–300) interacted with Paxillin, but USP13(301–624), USP13(301–863), and USP13(625–863) could not interact with Paxillin (Fig. 6k), indicating that 1aa–300aa of USP13 is involved in USP13-Paxillin interaction. Furthermore, Paxillin and Paxillin(Y118A) interacted with USP13, but Paxillin(Y31A) failed to associate with USP13 (Fig. 6l), implicating that Paxillin Y31 phosphorylation is important for USP13-Paxillin interaction.

To determine the role of USP13 in the regulation of NLRP3 ubiquitination, we generated HEK293T cells stably expressing sh-USP13. The level of NLRP3 ubiquitination was promoted by sh-USP13 (Fig. 6m), facilitated by Spautin-1 (an inhibitor of deubiquitinating enzyme activity of USP13) (Fig. 6n), attenuated by Paxillin, whereas Paxillin-mediated attenuation of NLRP3 ubiquitination was repressed by Spautin-1 (Fig. 6o), indicating that USP13 is required for NLRP3 deubiquitination. Moreover, IL-1β secretion, IL-1β maturation, and Casp-1 cleavage induced by ATP were repressed by Spautin-1 (Fig. 6p, q), suggesting that USP13 deubiquitinating enzyme activity is critical for ATP-induced NLRP3 inflammasome activation. Taken together, these data demonstrate that USP13 is essential for Paxillin-mediated deubiquitination of NLRP3 upon ATP treatment.

### ATP induces Paxillin and NLRP3 membrane migration to facilitate P2X7R-Paxillin-NLRP3 complex formation

**Fig. 7:**
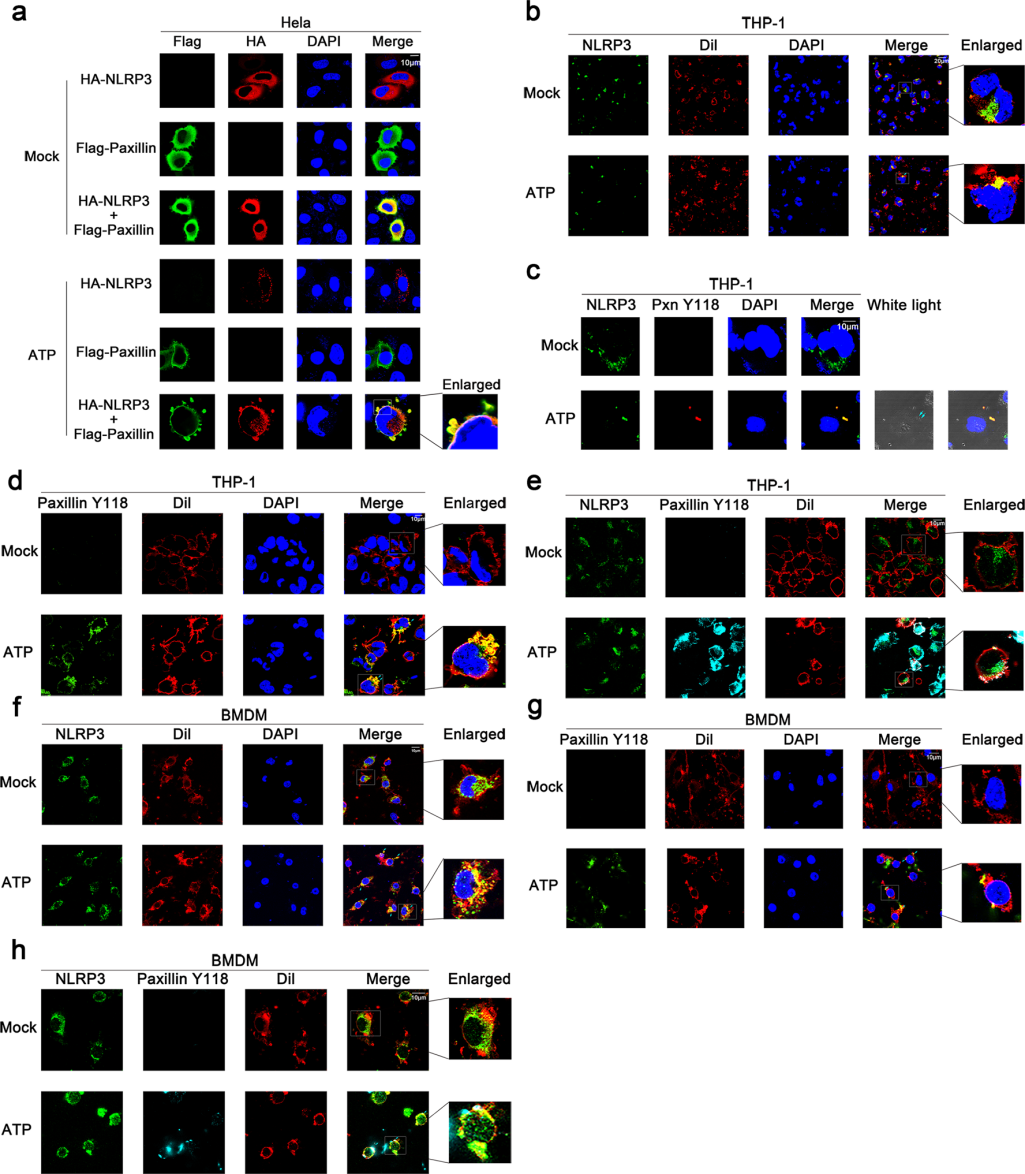
ATP induces Paxillin and NLRP3 localization near the plasma membrane. **a** Hela cells were transfected with pFlag-Paxillin and pHA-NLRP3, or co-transfected with pFlag-Paxillin and pHA-NLRP3, and treated with ATP (5 mM) for 2 h. Subcellular localization of Flag-Paxillin (green), HA-NLRP3 (red), and the nucleus marker DAPI (blue) was examined by confocal microscopy. **b** TPA-differentiated THP-1 macrophages were treated with ATP (5 mM) for 2 h. Subcellular localization of NLRP3 (green), the membrane marker Dil (red), and DAPI was examined by confocal microscopy. **c** TPA-differentiated THP-1 macrophages were treated with ATP (5 mM) for 2 h. Subcellular localization of NLRP3 (green), Paxillin(Y118) (red), DAPI (blue), and white light was examined by confocal microscopy. **d** TPA-differentiated THP-1 macrophages were treated with ATP (5 mM) for 2 h. Subcellular localization of Paxillin(Y118) (green), the membrane marker Dil (red), and DAPI (blue) was examined by confocal microscopy. **e** TPA-differentiated THP-1 macrophages were treated with ATP (5 mM) for 2 h. Subcellular localization of NLRP3 (green), Paxillin(Y118) (cyan), and the membrane marker Dil (red) was examined by confocal microscopy. **f** LPS-primed BMDMs were stimulated by ATP (5 mM) for 30 min. Subcellular localization of NLRP3 (green), the membrane marker Dil (red), and DAPI (blue) was examined by confocal microscopy. **g** LPS-primed BMDMs were stimulated by ATP (5 mM) for 30 min. Subcellular localization of Paxillin(Y118) (green), the membrane marker Dil (red), and DAPI (blue) was examined by confocal microscopy. **h** LPS-primed BMDMs were stimulated by ATP (5 mM) for 30 min. Subcellular localization of NLRP3 (green), Paxillin(Y118) (cyan), and the membrane marker Dil (red) was examined by confocal microscopy

As Paxillin is a scaffold for the recruitment of proteins into a complex apposing to the plasma membrane [2], and Paxillin facilitates the P2X7R-Paxillin-NLRP3 complex assembly, we thus explored whether Paxillin recruits NLRP3 to the plasma membrane. In Hela cells, NLRP3 alone and Paxillin alone diffusely distributed in the cytosol in the absence of ATP; NLRP3 forms small spots in the cytosol and Paxillin localized in the membrane, whereas NLRP3 and Paxillin together co-localized and formed membrane blebbing in the presence of ATP (Fig. 7a), similar to that described previously [25]. In TPA-differentiated THP-1 macrophages, endogenous NLRP3 was diffusely distributed in the cytosol without ATP but formed spots in the membrane as indicated by Dil (red) (a dye of cell membrane) in the presence of ATP (Fig. 7b); endogenous phosphorylated Paxillin was hardly detected in the absence of ATP, whereas it was co-localized with NLRP3 in the membrane upon ATP treatment (Fig. 7c); endogenous phosphorylated Paxillin was hardly detected without ATP, whereas it was detected in the membrane upon ATP treatment (Fig. 7d); and notably, endogenous NLRP3 and phosphorylated Paxillin co-localized and formed spots in the membrane upon ATP treatment (Fig. 7e). Moreover, in LPS-primed BMDMs, endogenous NLRP3 was diffusely distributed in the cytosol without ATP, but localized and formed spots in the membrane upon ATP treatment (Fig. 7f); endogenous phosphorylated Paxillin was hardly detected without ATP, but localized in the membrane after ATP treatment (Fig. 7g); and endogenous NLRP3 and phosphorylated Paxillin co-localized and formed spots in the membrane after treated with ATP (Fig. 7h). Collectively, these results suggest that ATP induces NLRP3 and Paxillin translocation from the cytosol to the plasma membrane.

**Fig. 8:**
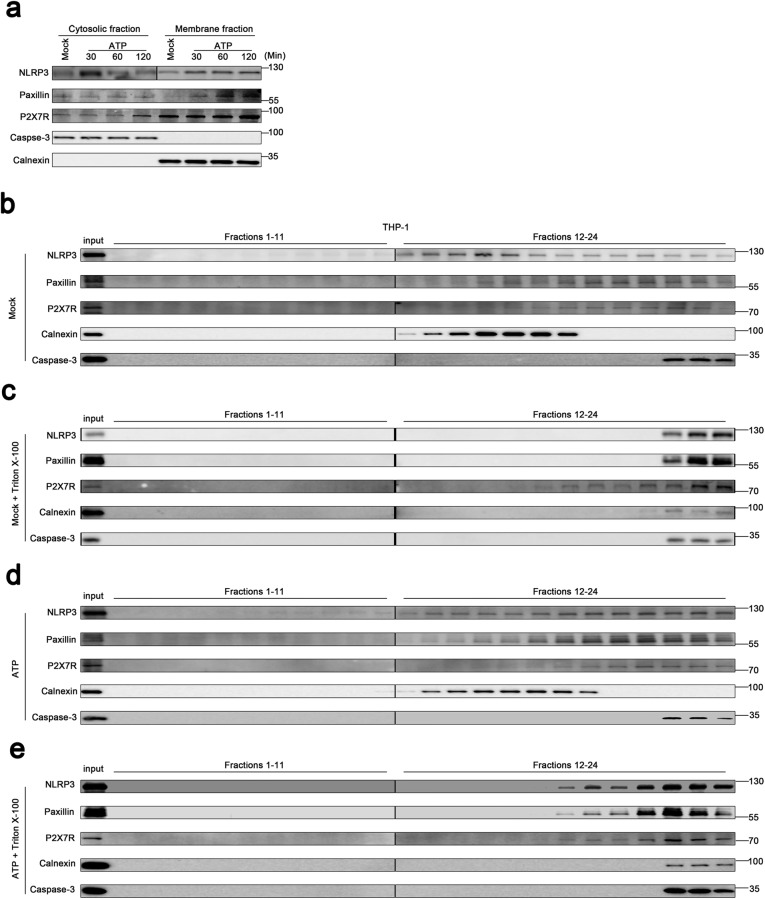
ATP induces Paxillin and NLRP3 membrane migration to facilitate the formation of the P2X7R-Paxillin-NLRP3 complex. **a** Subcellular fractionation of TPA-differentiated THP-1 macrophages in the presence of ATP (5 mM) for 30, 60, and 120 min. The protein levels of NLRP3, Paxillin, P2X7R, Caspase-3, and Calnexin in the cytosolic fraction (left) and membrane fraction (right) were determined by Western blot. **b**–**e** Membrane flotation assays of TPA-differentiated THP-1 macrophages post-nuclear lysates on a 10–45% Optiprep gradient under the treatment of ATP (5 mM) for 30 min in the presence or absence of 1% Triton X-100. The protein level of NLRP3, Paxillin, P2X7R, Caspase-3, and Calnexin was determined by Western blot in each 24 fractions and used as the input

The distribution of NLRP3 in TPA-differentiated THP-1 macrophages upon ATP treatment was examined. Fractionation fidelity was verified by Caspase-3 in the cytosolic fraction and Calnexin in the membrane fraction. Without ATP treatment, NLRP3, Paxillin, and P2X7R localized in the cytosolic fraction and membrane fraction, Caspase-3 localized in the cytosolic fraction, and Calnexin distributed in membrane fraction; upon ATP treatment, NLRP3, Paxillin, and P2X7R distributed in the membrane fraction (Fig. 8a), suggesting that NLRP3 and Paxillin migrate from the cytosol to membrane upon ATP stimulation. Membrane flotation assay can indicate that membrane-bound organelles migrate from dense to light fractions [26], and membrane-bound organelles are sensitive to Triton X-100 except for the plasma membrane [27, 28]. We thus performed membrane flotation assays on Optiprep gradients bottom-loaded with lysate supernatants of TPA-differentiated THP-1 macrophages. Without treatments, NLRP3 floated from the bottom fractions (21–24) into the membrane fractions (12–20), Paxillin floated from the bottom fractions (21–24) into the membrane fractions (14–20), and P2X7R floated from the bottom fractions (21–24) into the lighter fractions (15–20), while Calnexin remained in the lighter membrane fraction (12–18) and Caspase-3 stayed in the bottom fractions (22–24) (Fig. 8b). Notably, after Triton X-100 treatment, NLRP3, Paxillin, Calnexin, and Caspase-3 remained in the bottom fractions (22–24), whereas P2X7R floated from the bottom fractions (22–24) into the lighter fractions (17–21) (Fig. 8c). Notably, upon ATP stimulation, NLRP3 floated from the bottom fractions (21–24) into the membrane fractions (12–20), Paxillin floated from the bottom fractions (21–24) into the lighter fractions (13–20), and P2X7R floated from the bottom fractions (21–24) into the membrane fractions (17–20), while Calnexin remained in the membrane fraction (12–19) and Caspase-3 retained in the bottom fractions (22–24) (Fig. 8d). Interestingly, upon ATP stimulation and Triton X-100 treatment, NLRP3, Paxillin, and P2X7R floated from the bottom fractions (21–24) into the membrane fractions (18–20), Calnexin retained the bottom fractions (21–24), and Caspase-3 remained in the bottom fractions (22–24) (Fig. 8e). Together, the results demonstrate that NLRP3, Paxillin, and P2X7R colocalized on the plasma membrane upon ATP treatment and suggest that ATP induces Paxillin and NLRP3 membrane migration to facilitate P2X7R-Paxillin-NLRP3 complex formation.

### Paxillin is required for ATP- and Nigericin-induced NLRP3 inflammasome activation

**Fig. 9:**
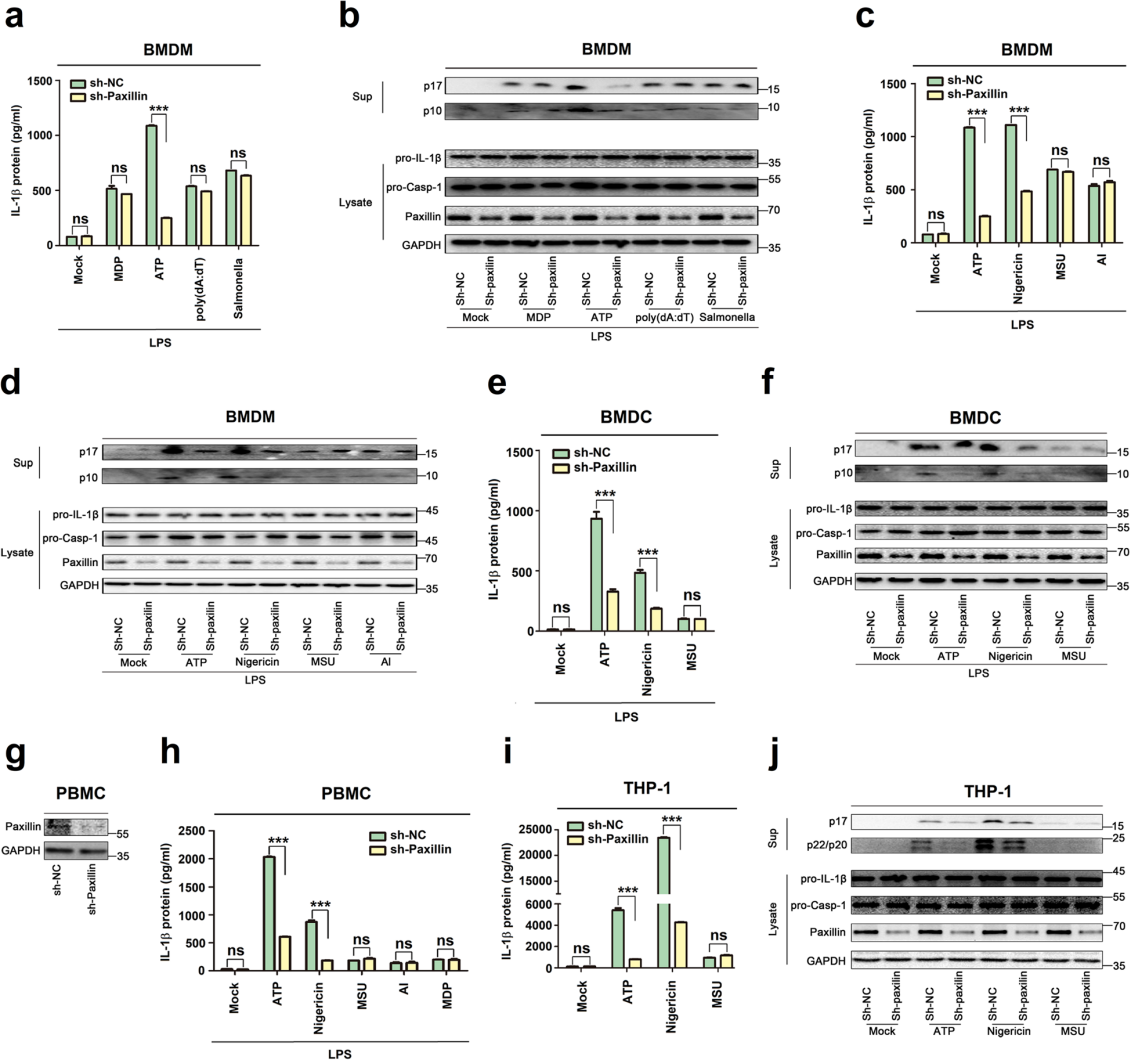
Paxillin is required for ATP- and Nigericin-induced NLRP3 inflammasome activation. **a**, **b** BMDMs prepared from C57BL/6 mice bone marrow were infected by lentivirus that express sh-Paxillin for 3 days. LPS-primed BMDMs were stimulated by MDP (50 μg/ml) for 6 h, ATP (5 mM) for 30 min, poly (dA:dT) (5 μg/ml) for 6 h, or Salmonella for 4 h. **c**, **d** LPS-primed BMDMs were stimulated by ATP (5 mM) for 30 min, Nigericin (2 μM) for 2 h, MSU (100 μg/ml) for 6 h, or Alum crystals (200 mg/ml) for 6 h. **e**, **f** BMDCs prepared from C57BL/6 mice bone marrow were infected by lentivirus that express sh-Paxillin for 3 days. LPS-primed BMDCs were stimulated by ATP (5 mM) for 30 min, Nigericin (2 μM) for 2 h, or MSU (100 μg/ml) for 6 h. IL-1β levels in supernatants were determined by ELISA (**a**, **c**, **e**). Mature IL-1β (p17) and cleaved Casp-1 (p10) in supernatants as well as Paxillin, pro-IL-1β, and pro-Casp-1 in lysates were determined by Western blot (**b**, **d**, **f**). **g**, **h** PBMCs isolated from healthy individuals were infected by lentivirus that express sh-Paxillin for 2 days. Before stimulation, PBMCs were treated with LPS (1 μg/ml) for 6 h, and PBMCs were then stimulated by ATP (5 mM) for 30 min, Nigericin (2 μM) for 2 h, MSU (100 μg/ml) for 6 h, Alum crystals (200 mg/ml) for 6 h, or MDP (50 μg/ml) for 6 h. Paxillin in lysates was determined by Western blot (**g**). IL-1β levels in supernatants were determined by ELISA (**h**). **i**, **j** THP-1 cells stably expressing sh-NC or sh-Paxillin were generated and differentiated into macrophages, which were then treated with ATP (5 mM) for 2 h, Nigericin (2 μM) for 2 h, or MSU (100 μg/ml) for 6 h. IL-1β levels in supernatants were determined by ELISA (**i**). Mature IL-1β (p17) and cleaved Casp-1 (p22/p20) in supernatants and Paxillin, pro-IL-1β, and pro-Casp-1 in lysates were determined by Western blot (**j**). Data shown are means ± SEMs; ***p < 0.0001. ns, no significance

At least three NLRP family members (NLRP1, NLRP3, and NLRC4/IPAF) and one HIN-200 family member (absent in melanoma 2 (AIM2)) have been reported to exhibit inflammasome activity [1]. The NLRP1 inflammasome is activated by mycobacterial DNA-binding protein (MDP) [29]. The NLRP3 inflammasome is induced by ATP [20], monosodium urate (MSU) [30], Nigericin [20], and Alum (Al) [31]. The NLRC4 inflammasome is stimulated by bacteria [32]. AIM2 senses cytosolic double-stranded DNA (dsDNA) [33]. Here, we determined the biological roles of Paxillin in the regulation of the four inflammasomes. BMDMs stably expressed sh-Paxillin were generated by infected with lentivirus expressing sh-Paxillin. The stable BMDMs were treated with four inflammasome activators, MDP for NLRP1 inflammasome, ATP for NLRP3 inflammasome, dA:dT for AIM2 inflammasome, and Salmonella for NLRC4 inflammasome. ATP-induced IL-1β secretion, IL-1β maturation, and Caspase-1 cleavage were attenuated by sh-Paxillin, whereas MDP-, dA:dT-, or Salmonella-induced IL-1β secretion; IL-1β maturation; and Caspase-1 cleavage were not affected by sh-Paxillin (Fig. 9a, b), demonstrating that Paxillin is essential for the NLRP3 inflammasome activation.

**Fig. 10:**
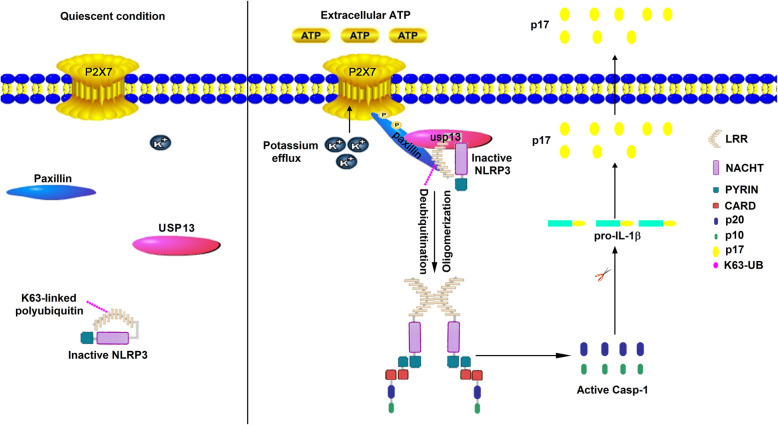
Paxillin regulates ATP-induced activation of P2X7R receptor and NLRP3 inflammasome by formatting the P2X7R-Paxillin-NLRP3 complex. In the quiescent condition, the inactive, Paxillin and USP13 distribute in the cytoplasm (left). However, in response to extracellular ATP, Paxillin is a molecular adaptor protein, which recruits usp13 and NLRP3 at the plasma membrane for the activation of the NLRP3 inflammasome for efficient processing of P2X7R (right)

Stable BMDMs were then treated with ATP, Nigericin, monosodium urate (MSU), and alum (Al). The results showed that ATP- and Nigericin-induced IL-1β secretion, IL-1β maturation, and Caspase-1 cleavage were attenuated by sh-Paxillin, whereas MSU- or Al-mediated IL-1β secretion, IL-1β maturation, and Caspase-1 cleavage were not affected by sh-Paxillin (Fig. 9c, d). In addition, mouse bone marrow dendritic cells (BMDCs) stably expressed sh-Paxillin were constructed. Stable BMDCs were treated with ATP, Nigericin, and MSU. Similarly, ATP- and Nigericin-induced IL-1β secretion, IL-1β maturation, and Caspase-1 cleavage were reduced by sh-Paxillin, but MSU-mediated IL-1β secretion, IL-1β maturation, and Casp-1 cleavage were not affected by sh-Paxillin (Fig. 9e, f). Moreover, human peripheral blood mononuclear cells (PBMCs) stably expressed sh-Paxillin were generated. The stable PBMCs were treated with ATP, Nigericin, MSU, Al, or MDP. ATP- and Nigericin-induced IL-1β secretion was downregulated by sh-Paxillin, but MSU-, Al-, or MDP-mediated IL-1β secretion was not affected by sh-Paxillin, and the level of Paxillin was attenuated by sh-Paxillin (Fig. 9g, h). Finally, THP-1 cells stably expressed sh-Paxillin were generated. The stable THP-1 cells were differentiated into macrophages, which were then treated with ATP, Nigericin, or MSU. ATP- and Nigericin-induced IL-1β secretion, IL-1β maturation, and Caspase-1 cleavage were attenuated by sh-Paxillin, but MSU-mediated IL-1β secretion, IL-1β maturation, and Casp-1 cleavage were not affected by sh-Paxillin (Fig. 9i, j). Collectively, these results demonstrate that Paxillin is essential for ATP- and Nigericin-induced NLRP3 inflammasome activation. Taken together, we demonstrate that Paxillin plays key roles in ATP-induced activation of the P2X7 receptor and NLRP3 inflammasome by formatting the P2X7R-Paxillin-NLRP3 complex (Fig. 10).

## Discussion

NLRP3 inflammasome activation requires P2X7 receptor stimulation mediated by ATP [5–7]. Upon ATP treatment, P2X7R induces transmembrane K^+^ ion efflux and Pannex-1 hemichannel to form large pore for inflammasome activation [25, 34]. However, the molecule connecting the P2X7 receptor and NLRP3 inflammasome has not been revealed. This study identifies that Paxillin plays key roles in ATP-induced activation of the P2X7 receptor and NLRP3 inflammasome by promoting the formation of the P2X7R-Paxillin-NLRP3 complex. The primary function of Paxillin is a molecular adapter or scaffold protein that provides multiple docking sites at the plasma membrane for processing of Integrin- and growth factor-mediated signals [12]. Initially, we demonstrated a direct interaction between Paxillin and NLRP3. The function and localization of Paxillin are tightly modulated by phosphorylation [35]. Phosphorylations at Y31 and Y118 sites in FAK- and Src-dependent manners [36] are essential for the interaction between Paxillin and downstream effectors, such as ERK [11] and Crk2 [37]. Tyrosine phosphorylation of Paxillin regulates the assembly and turnover of adhesion complexes [38]. Here, we demonstrate that ATP-induced Paxillin Y118 phosphorylation is essential for Paxillin-NLRP3 interaction and Paxillin promotes NLRP3 inflammasome activation upon ATP treatment.

Extracellular ATP stimulates the P2X7R ATP-gated ion channel, triggers K^+^ efflux, and induces gradual recruitment of the Pannexin-1 membrane pore, leading to inflammasome activation [8, 20, 22]. Interestingly, Paxillin interacts with P2X7R, ATP promotes P2X7R-Paxillin interaction, Paxillin Y31 phosphorylation is required for such interaction, and P2X7R K30 is essential for P2X7R-Paxillin association. Thus, we showed that ATP stimulates P2X7R-Paxillin interaction, induces K^+^ efflux, activates Paxillin phosphorylation, and promotes Paxillin-NLRP3 interaction, and suggest that Paxillin plays a key role in the P2X7R-Paxillin-NLRP3 complex assembly. Paxillin is a downstream adaptor of Integrin and functions as a scaffold for protein recruitment into a complex [9]. Our finding showed a new function of Paxillin in the recruitment of proteins involved in ATP-induced activation of the P2X7 receptor and NLRP3 inflammasome.

The molecular mechanism underlying the function of Paxillin in the activation of the P2X7 receptor and NLRP3 inflammasome is determined. As NLRP3 deubiquitination is important for the inflammasome activation [24], the roles of ATP and Paxillin in NLRP3 deubiquitination were evaluated. Notably, Paxillin facilitates the removal of NLRP3 K63-linked ubiquitination at K689, and ATP and K^+^ efflux are critical for this regulation. The importance of K^+^ efflux in NLRP3 inflammasome activation has been revealed [39]. The drop of K^+^ concentration triggers NLRP3 inflammasome, whereas a high concentration of K^+^ blocks NLRP3 inflammasome [40]. However, the molecular mechanism between decreased levels of intracellular K^+^ and NLRP3 activation remains largely unknown. A previous study has shown that intracellular K^+^ reduction mediated by P2X7R enhances NLRP3 interaction with NEK7 [41]. The present study identifies that Paxillin is essential for ATP-induced NLRP3 inflammasome activation and for NLRP3 recruitment in the plasma membrane.

Post-translational modifications (PTMs), including ubiquitination [24], phosphorylation [42], and Sumoylation [43] are critical for inflammasome activation. NLRP3 deubiquitination is required for NLRP3 inflammasome activation, and pharmacological inhibition of NLRP3 deubiquitination impairs the inflammasome activation [24]. ATP and Nigericin induce NLRP3 deubiquitination and inflammasome activation [44]. We demonstrate that ATP-induced Paxillin Y118 phosphorylation promotes the removal of NLRP3 K63-linked ubiquitination at K689. Additionally, we discovered that USP13 is the deubiquitinating enzyme required for Paxillin-mediated NLRP3 deubiquitination. USP13 interacts with Paxillin and NLRP3, phosphorylation of Paxillin at Y31 is important for Paxillin-USP13 interaction, and USP13 is essential for Paxillin-mediated NLRP3 deubiquitination upon ATP treatment.

ATP induces Paxillin-containing membrane protrusions [45], and Paxillin acts as a scaffold for protein recruitments into a complex apposing to the plasma membrane [9]. Confocal microscopy analysis shows that ATP induces the translocation of endogenous NLRP3 and endogenous Paxillin from the cytosol to the plasma membrane. Membrane flotation assay indicates that NLRP3, Paxillin, and P2X7R colocalize to the plasma membrane upon ATP treatment. Therefore, we suggest that ATP induces Paxillin and NLRP3 membrane migration to facilitate P2X7R-Paxillin-NLRP3 complex formation.

## Conclusions

The biological role of Paxillin in NLRP3 inflammasome activation is determined in this study. We demonstrate that Paxillin is essential for ATP-induced NLRP3 inflammasome activation, including IL-1β secretion, IL-1β maturation, and Caspase-1 cleavage. After the response to tissue damage and cellular stress, ATP is released to enhance tissue repair and promote the recruitment of immune phagocytes and dendritic cells [46]. ATP-P2X7 receptor signaling is involved in many pathological conditions, including infectious diseases, inflammatory diseases, and neurodegenerative disorders [47]. We demonstrate that Paxillin is a molecular adaptor that recruits USP13 and NLRP3 on the plasma membrane for NLRP3 inflammasome activation, and thereby may act as a potential target of therapeutics to inflammatory diseases.

## Methods

### Animal study

Mouse BMDCs were differentiated from fresh bone marrow cells of C57BL/6 WT mice in RPMI 1640 medium containing 10% heat-inactive fetal bovine serum (FBS) in the presence of granulocyte-macrophage colony-stimulating factor (GM-CSF) in six-well plates for 6 days. The culture medium was replaced every other day.

Mouse BMDMs were differentiated from fresh bone marrow cells of C57BL/6 WT mice. The bone marrow cells were incubated in six-well plates for 6 days with 10% L929-conditioned and 10% heat-inactive FBS in RPMI 1640 medium. The culture medium was replaced every 2 days. The animal study was approved by the Institutional Review Board of the College of Life Sciences, Wuhan University, and was conducted in accordance with the guidelines for the protection of animal subjects (permit numbers: 2018–008).

### Cell lines and cultures

Hela and human embryonic kidney cells (HEK293T) were purchased from American Type Culture Collection (ATCC) (Manassas, VA, USA). The human acute monocytic leukemia cell line (THP-1) was a gift from Dr. Jun Cui of State Key Laboratory of Biocontrol, School of Life Sciences, Sun Yat-sen University, Guangzhou 510275, China. THP-1 cells were cultured in RPMI 1640 medium supplemented with 10% heat-inactivated FBS, 100 U/ml penicillin, and 100 μg/ml streptomycin sulfate. Hela and HEK293T cells were cultured in Dulbecco’s modified Eagle’s medium (DMEM) purchased from Gibco (Grand Island, NY, USA) supplemented with 10% FBS, 100 U/ml penicillin, and 100 μg/ml streptomycin sulfate. Hela, HEK293T, and THP-1 cells were maintained in an incubator at 37 °C in a humidified atmosphere of 5% CO_2_.

### Reagents

Phorbol-12-myristate-13-acetate (TPA) (P1585), OptiPrepTM (D1556), and DMSO (D8418) were purchased from Sigma-Aldrich (St. Louis, MO, USA). RPMI 1640 and Dulbecco’s modified Eagle’s medium (DMEM) were obtained from Gibco (Grand Island, NY, USA). Lipopolysaccharide (LPS), adenosine triphosphate (ATP) (987-65-5), Nigericin (28643-80-3), dA:dT (86828-69-5), MDP (53678-77-6), Glybenclamide (10238-21-8), MSU (1198-77-2), Alum Crystals (7784-24-9), and Ac-YVAD-cmk (178603-78-6) were obtained from InvivoGen Biotech Co., Ltd. (San Diego, CA, USA). A438079 (899431-18-6), AZ10606120 (607378-18-7), and BAPTA-AM (126150-97-8) were purchased from Tocris Bioscience. Antibody against Flag (Catalog No. F3165), HA (Catalog No. H6908), and monoclonal mouse anti-GAPDH (Catalog No. G9295) were purchased from Sigma (St. Louis, MO, USA). Monoclonal rabbit anti-NLRP3 (D2P5E) (Catalog No. 13158), ubiquitin mouse mAb (P4D1) (Catalog No. 3936), monoclonal rabbit anti-K63-linkage-specific polyubiquitin (D7A11) (Catalog No. 5621), monoclonal rabbit anti-Caspase-3 (13809S) (Catalog No. 13809), monoclonal rabbit anti-P2X7R (E1E8T) (Catalog No. 13809), monoclonal rabbit anti-p-Paxillin (Tyr118) (Catalog No. 2541), monoclonal rabbit anti-calnexin (C5C9) (Catalog No. 2679), monoclonal rabbit anti-IL-1β (D3U3E) (Catalog No. 12703), IL-1β mouse mAb (3A6) (Catalog No. 12242), and monoclonal rabbit anti-Caspase-1 (Catalog No. 2225) were purchased from Cell Signaling Technology (Beverly, MA, USA). Monoclonal mouse anti-ASC (Catalog No. sc-271054), polyclonal rabbit anti-caspase-1 p10 (Catalog No. sc-515), and polyclonal rabbit anti-IL-1β (Catalog No. sc-7884) were purchased from Santa Cruz Biotechnology (Santa Cruz, CA, USA). Polyclonal goat anti-mouse IL-1β (p17) (Catalog No. AF-401-NA) was from R&D Systems. Monoclonal mouse anti-Paxillin (Catalog No. 7019741) was purchased from BD Biosciences. Polyclonal rabbit anti-p-Paxillin (Tyr31) (Catalog No. 1690606A) was purchased from Life Technologies. Monoclonal mouse anti-NLRP3 (Catalog No. AG-20B-0014-C100) was purchased from Adipogen to detect endogenous NLRP3 in THP-1 cells, BMDCs, and BMDMs. Lipofectamine 2000, normal rabbit IgG, and normal mouse IgG were purchased from Invitrogen Corporation (Carlsbad, CA, USA).

### Plasmid construction

The cDNAs encoding human paxillin, NLRP3, ASC, pro-Casp-1, and IL-1β were obtained by reverse transcription of total RNA from TPA-differentiated THP-1 cells, followed by PCR using specific primers. The cDNAs were sub-cloned into pcDNA3.1(+) and pcagg-HA vector. The pcDNA3.1(+)-3×Flag vector was constructed from the pcDNA3.1(+) vector through inserting the 3×Flag sequence between the NheI and HindIII site. The following are the primers used in this study.

Flag-NLRP3: 5′-CGCGGATCCATGAAGATGGCAAGCACCCGC-3′, 5′-CCGCTCGAGCTACCAAGAAGGCTCAAAGAC-3′; Flag-ASC: 5′-CCGGAATTCATGGGGCGCGCGCGCGACGCCAT-3′, 5′-CCGCTCGAGTCAGCTCCGCTCCAGGTCCTCCA-3′; Flag-Casp-1: 5′-CGCGGATCCATGGCCGACAAGGTCCTGAAG-3′, 5′-CCGCTCGAGTTAATGTCCTGGGAAGAGGTA-3′; Flag-paxillin: 5′-CCGGAATTCTATGGACGACCTCGACGCCCT-3′, 5′-CCGCTCGAGCTAGCAGAAGAGCTTGAGGAA-3′; Myc-paxillin: 5′-CCGGAATTCTATGGACGACCTCGAC-3′, 5′-CCGCTCGAGCTAGCAGAAGAGCTTG-3′; Myc-paxillin(y118a): 5′-GTGAGGAGGAGCACGTCGCAAGCTTCCCCAACAAGCAGAA-3′, 5′-ATTTCTGCTTGTTGGGGAAGCTTGCGACGTGCTCCTCCTC-3′; HA-paxillin: 5′-CCGGAATTCATGGACGACCTCGACGCCCTG-3′, 5′-CGCTCGAGGCAGAAGAGCTTGAGGAAGCA-3′; HA-paxillin(y31a): 5′-GCCTGTGTTCTTGTCGGAGGAGACCCCCGCATCATACCCA-3′, 5′-GTGTGGTTTCCAGTTGGGTATGATGCGGGGGTCTCCTCCG-3′; HA-paxillin(y118a): 5′-GTGAGGAGGAGCACGTCGCAAGCTTCCCCAACAAGCAGAA-3′, 5′-ATTTCTGCTTGTTGGGGAAGCTTGCGACGTGCTCCTCCTC-3′; HA-NLRP3: 5′-TACGAGCTCATGAAGATGGCAAGCACCCGC-3′, 5′-CCGCTCGAGCCAAGAAGGCTCAAAGACGAC-3′; pGEX-6p-1-paxillin: 5′-CCGGAATTCATGGACGACCTCGACGCCCTG-3′, 5′-CGCTCGAGGCAGAAGAGCTTGAGGAAGCA-3′; pGEX-6p-1-LRR: 5′-CGCGGATCCATGTCTCAGCAAATCAGGCTG-3′, 5′-CCGCTCGAGCTACCAAGAAGGCTCAAAGAC-3′; AD-paxillin: 5′-CCGGAATTCATGGACGACCTCGACGCCCTG-3′, 5′-CGCTCGAGGCAGAAGAGCTTGAGGAAGCA-3′; and pGBKT7-LRR: 5′-CGCGGATCCATATGTCTCAGCAAATCAGGC-3′, 5′-CCGCTCGAGCTACCAAGAAGGCTCAAAGAC-3′.

The P2X7R and USP13 truncates were cloned into pcDNA3.1(+), and the PYRIN, NACHT, and LRR domain of NLRP3 protein was cloned into pcDNA3.1(+) and pcaggs-HA vector using specific primers. The following are the primers used in this study.

Flag-PYRIN: 5′-AAAGGATCCATGAAGATGGCAAGCACCCGC-3′, 5′-CGGCTCGAGCTATAAACCCATCCACTCCTCTTC-3′; Flag-NACHT: 5′-AAAGGATCCCTGGAGTACCTTTCGAGAATCTC-3′, 5′-CCCCTCGAGCTAGATCTTGCAACTTAATTTCTTC-3′; Flag-LRR: 5′-AAAGGATCCTCTCAGCAAATCAGGCTGGAG-3′, 5′-CGGCTCGAGCTACCAAGAAGGCTCAAAGACG-3′; Flag-P2X7R: 5′-ATTGGTACCATGCCGGCCTGCTGCAGCTGCAGT-3′, 5′-CCGCTCGAGTCAGTAAGGACTCTTGAAGCCACT-3′; Flag-P2X7R(26–595): 5′-CAAGATATCATGTATGGCACCATTAAGTGG-3′, 5′-CCGCTCGAGTCAGTAAGGACTCTTGAAGCC-3′; Flag-P2X7R(47–595): 5′-AATGATATCATGAGTGACAAGCTGTACCAG-3′, 5′-CCGCTCGAGTCAGTAAGGACTCTTGAAGCC-3′; Flag-P2X7R(335–595): 5′-CCGGAATTCTATGGTGTACATCGGCTCAAC-3′, 5′-CCGCTCGAGTCAGTAAGGACTCTTGAAGCC-3′; Flag-P2X7R(356–595): 5′-CCGGAATTCTATGGACACTTACTCCAGTAA-3′, 5′-CCGCTCGAGTCAGTAAGGACTCTTGAAGCC-3′; Flag-P2X7R(27–595): 5′-CGGGGTACCATGGGCACCATTAAGTGGTTC-3′, 5′-CTAGTCTAGATCAGTAAGGACTCTTGAAGC-3′; Flag-P2X7R(29–595): 5′-CGGGGTACCATGATTAAGTGGTTCTTCCAC-3′, 5′-CTAGTCTAGATCAGTAAGGACTCTTGAAGC-3′; Flag-P2X7R(30–595): 5′-CGGGGTACCATGAAGTGGTTCTTCCACGTG-3′, 5′-CTAGTCTAGATCAGTAAGGACTCTTGAAGC-3′; Flag-P2X7R(31–595): 5′-CGGGGTACCATGTGGTTCTTCCACGTGATC-3′, 5′-CTAGTCTAGATCAGTAAGGACTCTTGAAGC-3′; Flag-P2X7R(32–595): 5′-CGGGGTACCATGTTCTTCCACGTGATCATC-3′, 5′-CTAGTCTAGATCAGTAAGGACTCTTGAAGC-3′; Flag-P2X7R(33–595): 5′-CGGGGTACCATGTTCCACGTGATCATCTTT-3′, 5′-CTAGTCTAGATCAGTAAGGACTCTTGAAGC-3′; Flag-P2X7R(34–595): 5′-CGGGGTACCATGCACGTGATCATCTTTTCC-3′, 5′-CTAGTCTAGATCAGTAAGGACTCTTGAAGC-3′; Flag-P2X7R(35–595): 5′-CGGGGTACCATGGTGATCATCTTTTCCTAC-3′, 5′-CTAGTCTAGATCAGTAAGGACTCTTGAAGC-3′; Flag-P2X7R(36–595): 5′-CGGGGTACCATGATCATCTTTTCCTACGTT-3′, 5′-CTAGTCTAGATCAGTAAGGACTCTTGAAGC-3′; Flag-P2X7R(k30a): 5′-AATTATGGCACCATTGCGTGGTTCTTCCACGTGAT-3′, 5′-ATGATCACGTGGAAGAACCACGCAATGGTGCCATA-3′; Flag-USP13: 5′-CGGGGTACCATGCAGCGCCGGGGCGCCCTG-3′, 5′-CCGCTCGAGTTAGCTTGGTATCCTGCGGTA-3′; Flag-usp13(1–300): 5′-CGGGGTACCATGCAGCGCCGGGGCGCCCTG-3′, 5′-CCGCTCGAGTTACCCATGCATATGAAGCAT-3′; Flag-usp13(301–624): 5′-CGGGGTACCATGACAGAGAATGGGCTCCAG-3′, 5′-CCGCTCGAGTTATTCCTCTCCTGGCTGTAA-3′; Flag-usp13(301–863): 5′-CGGGGTACCATGACAGAGAATGGGCTCCAG-3′, 5′-CCGCTCGAGTTAGCTTGGTATCCTGCGGTA-3′; Flag-usp13(625–863): 5′-CGGGGTACCATGGAACTTCCAGACATCAGC-3′, 5′-CCGCTCGAGTTAGCTTGGTATCCTGCGGTA-3′. HA-PYRIN: 5′-CCGGAATTCATGAAGATGGCAAGCACCCGC-3′, 5′-CCGCTCGAGTAAACCCATCCACTCCTCTTC-3′; HA-NACHT: 5′-CCGGAATTCATGCTGGAGTACCTTTCGAGA-3′, 5′-CCGCTCGAGGATCTTGCAACTTAATTTCTT-3′; and HA-LRR: 5′-ATCGAGCTCATGTCTCAGCAAATCAGGCTG-3′, 5′-CCGCTCGAGCCAAGAAGGCTCAAAGACGAC-3′.

### Lentivirus production and infection

The targeting sequences of shRNAs for the human Paxillin and mouse paxillin were as follows: human sh-Paxillin, 5′-CCCGACCTAATTGTCTTTGTT-3′; mouse sh-Paxillin, 5′-TCTGAACTTGACCGGCTGTTA-3′. A PLKO.1 vector encoding shRNA for GFP a negative control or a specific target molecule (Sigma-Aldrich) was transfected into HEK293T cells together with psPAX2 and pMD2.G with Lipofectamine 2000. Culture supernatants were harvested 36 and 60 h after transfection and then centrifuged at 2200 rpm for 15 min. THP-1 cells were infected with the supernatants containing lentiviral particles in the presence of 4 μg/ml polybrene (Sigma). After 48 h of culture, cells were selected by 1.5 μg/ml puromycin (Sigma) for 5 days. The BMDM and BMDC cells were infected with lentiviral particles for 1 day and treated with 1.5 μg/ml puromycin (Sigma) for 2 days. The results of each sh-RNA-targeted protein were detected by immunoblot analysis.

We using the 3*Flag sequence to replace the GFP protein in the pLenti CMV GFP Puro vector (Addgene, 658-5) for adding some restriction enzyme cutting site (XbaI-EcoRV-BstBI-BamHI) before the 3×Flag tag. Then the pLenti vector encoding paxillin protein was transfected into HEK293T cells together with psPAX2 and pMD2.G with Lipofectamine 2000. The following are the primers used in this study: pLenti-paxillin—5′-CTAGTCTAGAATGGACGACCTCGACGCCCT-3′, 5′-GATTTCGAAGCAGAAGAGCTTGAGGAAGCA-3′. Culture supernatants were harvested 36 and 60 h after transfection and then centrifuged at 2200 rpm for 15 min. THP-1 cells were infected with the supernatants containing lentiviral particles in the presence of 4 μg/ml polybrene (Sigma). After 48 h of culture, cells were selected by 1.5 μg/ml puromycin (Sigma) for 5 days. The results of the paxillin protein were detected by immunoblot analysis.

### Enzyme-linked immunosorbent assay

The concentrations of human IL-1β in culture supernatants were measured by the ELISA kit (BD Biosciences, San Jose, CA, USA). The mouse IL-1β ELISA Kit was purchased from R&D. All results were measured by two replicates.

### THP-1 macrophages stimulation

THP-1 cells were differentiated into macrophages with 60 nM phorbol-12-myristate-13-acetate (TPA) for 12–14 h, and cells were cultured for 24 h without TPA. And then, the differentiated cells were stimulated in 6-cm plates with Nigericin or ATP. Supernatants were collected for the measurement of IL-1β by ELISA. Cells were harvested for immunoblot analysis.

### Activated caspase-1 and mature IL-1β measurement

The supernatant of the cultured cells was collected for 1 ml in the cryogenic vials (Corning). The supernatant was frozen in − 80 °C for 4 h. The rotational vacuum concentrator machine which was purchase from Martin Christ was used for the freeze-drying. The drying product was dissolved in 100 μl PBS and mixed with SDS loading buffer for Western blotting analysis with antibodies for detection of activated caspase-1 (D5782 1:500, Cell Signaling), mature IL-1β (Asp116 1:500, Cell Signaling), polyclonal rabbit anti-caspase-1 p10 (sc-515), or polyclonal goat anti-mouse IL-1β (p17) (AF-401-NA). Adherent cells in each well were lysed with the lysis buffer described below, followed by immunoblot analysis to determine the cellular content of various proteins.

### Western blot analysis

HEK293T whole-cell lysates were prepared by lysing cells with buffer (50 mM Tris-HCl, pH 7.5, 300 mM NaCl, 1% Triton-X, 5 mM EDTA, and 10% glycerol). The TPA-differentiated THP-1 cell lysates were prepared by lysing cells with buffer (50 mM Tris-HCl, pH 7.5, 150 mM NaCl, 0.1% Nonidetp 40, 5 mM EDTA, and 10% glycerol). Protein concentration was determined by Bradford assay (Bio-Rad, Hercules, CA, USA). Cultured cell lysates (30 μg) were electrophoresed in an 8–12% SDS-PAGE gel and transferred to a PVDF membrane (Millipore, MA, USA). PVDF membranes were blocked with 5% skim milk in phosphate-buffered saline with 0.1% Tween 20 (PBST) before being incubated with the antibody. Protein bands were detected using a luminescent image analyzer (Fujifilm LAS-4000).

### Co-immunoprecipitation assays

HEK293T whole-cell lysates were prepared by lysing cells with buffer (50 mM Tris-HCl, pH 7.5, 300 mM NaCl, 1% Triton-X, 5 mM EDTA, and 10% glycerol). TPA-differentiated THP-1 cell lysates were prepared by lysing cells with buffer (50 mM Tris-HCl, pH 7.5, 150 mM NaCl, 0.1% Nonidetp40, 5 mM EDTA, and 10% glycerol). Lysates were immunoprecipitated with control mouse immunoglobulin G (IgG) (Invitrogen) or anti-Flag antibody (Sigma, F3165) with Protein-G Sepharose (GE Healthcare, Milwaukee, WI, USA).

### Confocal microscopy

HEK293T cells and Hela cells were transfected with plasmids for 24–36 h. Cells were fixed in 4% paraformaldehyde at room temperature for 15 min. After being washed three times with PBS, permeabilized with PBS containing 0.1% Triton X-100 for 5 min, washed three times with PBS, and finally blocked with PBS containing 5% BSA for 1 h. The cells were then incubated with the monoclonal mouse anti-Flag antibody (F3165, Sigma) and monoclonal rabbit anti-HA (H6908, Sigma) overnight at 4 °C, followed by incubation with FITC-conjugated donkey anti-mouse IgG (Abbkine) and Dylight 649-conjugated donkey anti-rabbit IgG (Abbkine) for 1 h. After washing three times, cells were incubated with DAPI solution for 5 min and then washed three more times with PBS. Finally, the cells were analyzed using a confocal laser scanning microscope (Fluo View FV1000; Olympus, Tokyo, Japan).

### GST pull-down assays

The plasmids pGEX6p-1-paxillin and pGEX6p-1-LRR were transfected into *Escherichia coli* strain BL21. After growing in LB medium at 37 °C until the OD600 reached 0.6–0.8, isopropyl β-d-1-thiogalactopyranoside (IPTG) was added to a final concentration of 1 mM and the cultures grew for an additional 4 h at 37 °C for GST-Paxillin and GST-LRR protein. And then, the GST protein, GST-paxillin protein, and GST-LRR protein were purified from *E. coli* bacteria. For GST-Paxillin pull-down assay, glutathione-Sepharose beads (Novagen) were incubated with GST-Paxillin or GST protein. After washed with phosphate-buffered saline (PBS), these beads were incubated with cell lysates from HEK293T which were transfected with plasmids encoding Flag-NLRP3 for 4 h at 4 °C. The precipitates were washed three times, boiled in 2× SDS loading buffer, separated by 10% SDS-PAGE, and immunoblotted with anti-GST and anti-Flag. It was the same for the GST-LRR pull-down assay.

### Yeast two-hybrid analyses

*Saccharomyces cerevisiae* strain AH109 and control vectors pGADT7, pGBKT7, pGADT7-T, pGBKT7-Lam, and pGBKT7-p53 were purchased from Clontech (Mountain View, CA, USA). Yeast strain AH109 was co-transformed with the combination of the pGADT7 and the pGBKT7 plasmids. Transformed yeast cells containing both plasmids were first grown on SD-minus Trp/Leu plates (DDO) to maintain the two plasmids and then were sub-cloned replica plated on SD-minus Trp/Leu/Ade/His plate (QDO).

### Subcellular fractionation

To separate cell membranes from the soluble cellular components, cells were washed once in hypotonic buffer (10 mM Tris-HCl, pH 7.4, 10 mM KCl, and 1.5 mM MgCl) supplemented with a protease inhibitor cocktail (Roche), incubated on ice in a hypotonic buffer, and lysed by Dounce homogenization. Lysates were centrifuged at 4 °C for 5 min at 2500×*g* to get the supernatants. Supernatants were centrifuged at 100,000×*g* for 1 h at 4 °C. The resultant pellets (membrane fraction) were resuspended in lysis buffer 1/5 volumes equal to those of the supernatants (cytosolic fraction), stored with the addition of 6× Laemmli buffer, and analyzed by Western blot.

For membrane flotation assays, post-nuclear supernatants were collected and described above, mixed with OptiprepTM-supplemented hypotonic buffer to yield a final concentration of 45% Optiprep at laid at the bottom of an OptiprepTM step gradient ranging from 10% (top) to 45% (bottom) and spun at 52,000×*g* for 90 min. The gradient was then fractionated into 24 fractions and analyzed by Western blot. For gradients run in the presence of Triton X-100, post-nuclear supernatants were mixed with a 10% Triton X-100 solution to achieve a final concentration of 1%.

### Statistical analyses

All experiments were reproducible and repeated at least three times with similar results. Parallel samples were analyzed for normal distribution using the Kolmogorov-Smirnov tests. Abnormal values were eliminated using a follow-up Grubbs test. Levene’s test for equality of variances was performed, which provided information for Student’s *t* tests to distinguish the equality of means. Means were illustrated using histograms with error bars representing the SD; a *p* value of < 0.05 was considered statistically significant.

## Supplementary Information

**Additional file 1.**

**Additional file 2:** **Fig. S1.** Full Western blots used for Fig. 1b, c, d, e. (a) Full Western blots used for Fig. 1b. (b) Full Western blots used for Fig. 1c. (c) Full Western blots used for Fig. 1d. (d) Full Western blots used for Fig. 1e. **Fig. S2.** Full Western blots used for Fig. 2b, d, e, f, g, h, i, j. (a) Full Western blots used for Fig. 2b. (b) Full Western blots used for Fig. 2d. (c) Full Western blots used for Fig. 2e. (d) Full Western blots used for Fig. 2f. (e) Full Western blots used for Fig. 2g. (f) Full Western blots used for Fig. 2h. (g) Full Western blots used for Fig. 2i. (h) Full Western blots used for Fig. 2j.**Fig. S3.** Full Western blots used for Fig. 3a, b, c, d, e, f, h, j, k. (a) Full Western blots used for Fig. 3a. (b) Full Western blots used for Fig. 3b. (c) Full Western blots used for Fig. 3c. (d) Full Western blots used for Fig. 3d. (e) Full Western blots used for Fig. 3e. (f) Full Western blots used for Fig. 3f. (g) Full Western blots used for Fig. 3h. (h) Full Western blots used for Fig. 3j. (i) Full Western blots used for Fig. 3k. **Fig. S4.** Full Western blots used for Fig. 4a, b, c, d, e, f, g. (a) Full Western blots used for Fig. 4a. (b) Full Western blots used for Fig. 4b. (c) Full Western blots used for Fig. 4c. (d) Full Western blots used for Fig. 4d. (e) Full Western blots used for Fig. 4e. (f) Full Western blots used for Fig. 4f. (g) Full Western blots used for Fig. 4g. **Fig. S5.** Full Western blots used for Fig. 5a, b, c, d, e, f. (a) Full Western blots used for Fig. 5a. (b) Full Western blots used for Fig. 5b. (c) Full Western blots used for Fig. 5c. (d) Full Western blots used for Fig. 5d. (e) Full Western blots used for Fig. 5e. (f) Full Western blots used for Fig. 5f. **Fig. S6.** Full Western blots used for Fig. 6a, b, c, d, e, f, g, h, j, k, l, m, n, o, q. (a) Full Western blots used for Fig. 6a. (b) Full Western blots used for Fig. 6b. (c) Full Western blots used for Fig. 6c. (d) Full Western blots used for Fig. 6d. (e) Full Western blots used for Fig. 6e. (f) Full Western blots used for Fig. 6f. (g) Full Western blots used for Fig. 6g. (h) Full Western blots used for Fig. 6h. (i) Full Western blots used for Fig. 6j. (j) Full Western blots used for Fig. 6k. (k) Full Western blots used for Fig. 6l. (l) Full Western blots used for Fig. 6m. (m) Full Western blots used for Fig. 6n. (n) Full Western blots used for Fig. 6o. (o) Full Western blots used for Fig. 6q. **Fig. S7.** Full Western blots used for Fig. 8a, b, c, d, e. (a) Full Western blots used for Fig. 8a. (b) Full Western blots used for Fig. 8b. (c) Full Western blots used for Fig. 8c. (d) Full Western blots used for Fig. 8d. (e) Full Western blots used for Fig. 8e. **Fig. S8.** Full Western blots used for Fig. 9b, d, f, g, j. (a) Full Western blots used for Fig. 9b. (b) Full Western blots used for Fig. 9d. (c) Full Western blots used for Fig. 9f. (d) Full Western blots used for Fig. 9g. (e) Full Western blots used for Fig. 9j.

## Data Availability

All data generated or analyzed during this study are included in this published article and its supplementary information files. Supporting data values and full blots are uploaded as supplementary information in Additional files.

## Abbreviations

NLRP3: NACHT, LRR, and PYD domain-containing protein 3
PRRs: Pattern recognition receptors
PAMPs: Pathogen-associated molecular patterns
DAMPs: Danger-associated molecular patterns
IL-1β: Interleukin-1β
ASC: Apoptosis-associated speck-like protein with CARD domain
ATP: Adenosine triphosphate
USP13: Ubiquitin-specific peptidase 13
Co-IP: Co-immunoprecipitation
GST: Glutathione S-transferase
BMDMs: Bone marrow-derived macrophages
AIM2: Absent in melanoma 2
MSU: Monosodium urate
Al: Alum
dsDNA: Double-stranded DNA
BMDCs: Mouse bone marrow dendritic cells
