# Supplementary material for: Paxillin mediates ATP-induced activation of P2X7 receptor and NLRP3 inflammasome
Source: BMC Biol. 2020 Nov 26;18:182. doi: 10.1186/s12915-020-00918-w (PMC7694937; doi:10.1186/s12915-020-00918-w)
Supplement: Supplementary file 2 — Additional file 2: Fig. S1. Full Western blots used for Fig. 1b, c, d, e. (a) Full Western blots used for Fig. 1b. (b) Full Western blots used for Fig. 1c. (c) Full Western blots used for Fig. 1d. (d) Full Western blots used for Fig. 1e. Fig. S2. Full Western blots used for Fig. 2b, d, e, f, g, h, i, j. (a) Full Western blots used for Fig. 2b. (b) Full Western blots used for Fig. 2d. (c) Full Western blots used for Fig. 2e. (d) Full Western blots used for Fig. 2f. (e) Full Western blots used for Fig. 2g. (f) Full Western blots used for Fig. 2h. (g) Full Western blots used for Fig. 2i. (h) Full Western blots used for Fig. 2j.Fig. S3. Full Western blots used for Fig. 3a, b, c, d, e, f, h, j, k. (a) Full Western blots used for Fig. 3a. (b) Full Western blots used for Fig. 3b. (c) Full Western blots used for Fig. 3c. (d) Full Western blots used for Fig. 3d. (e) Full Western blots used for Fig. 3e. (f) Full Western blots used for Fig. 3f. (g) Full Western blots used for Fig. 3h. (h) Full Western blots used for Fig. 3j. (i) Full Western blots used for Fig. 3k. Fig. S4. Full Western blots used for Fig. 4a, b, c, d, e, f, g. (a) Full Western blots used for Fig. 4a. (b) Full Western blots used for Fig. 4b. (c) Full Western blots used for Fig. 4c. (d) Full Western blots used for Fig. 4d. (e) Full Western blots used for Fig. 4e. (f) Full Western blots used for Fig. 4f. (g) Full Western blots used for Fig. 4g. Fig. S5. Full Western blots used for Fig. 5a, b, c, d, e, f. (a) Full Western blots used for Fig. 5a. (b) Full Western blots used for Fig. 5b. (c) Full Western blots used for Fig. 5c. (d) Full Western blots used for Fig. 5d. (e) Full Western blots used for Fig. 5e. (f) Full Western blots used for Fig. 5f. Fig. S6. Full Western blots used for Fig. 6a, b, c, d, e, f, g, h, j, k, l, m, n, o, q. (a) Full Western blots used for Fig. 6a. (b) Full Western blots used for Fig. 6b. (c) Full Western blots used for Fig. 6c. (d) Full Western blots used for Fig. 6d. (e) Full Western bl [file 12915_2020_918_MOESM2_ESM.docx]

**Paxillin Mediates ATP-induced Activation of P2X7 Receptor and NLRP3 Inflammasome**

**Supplementary Information**

**Supplementary Figures and Legends**


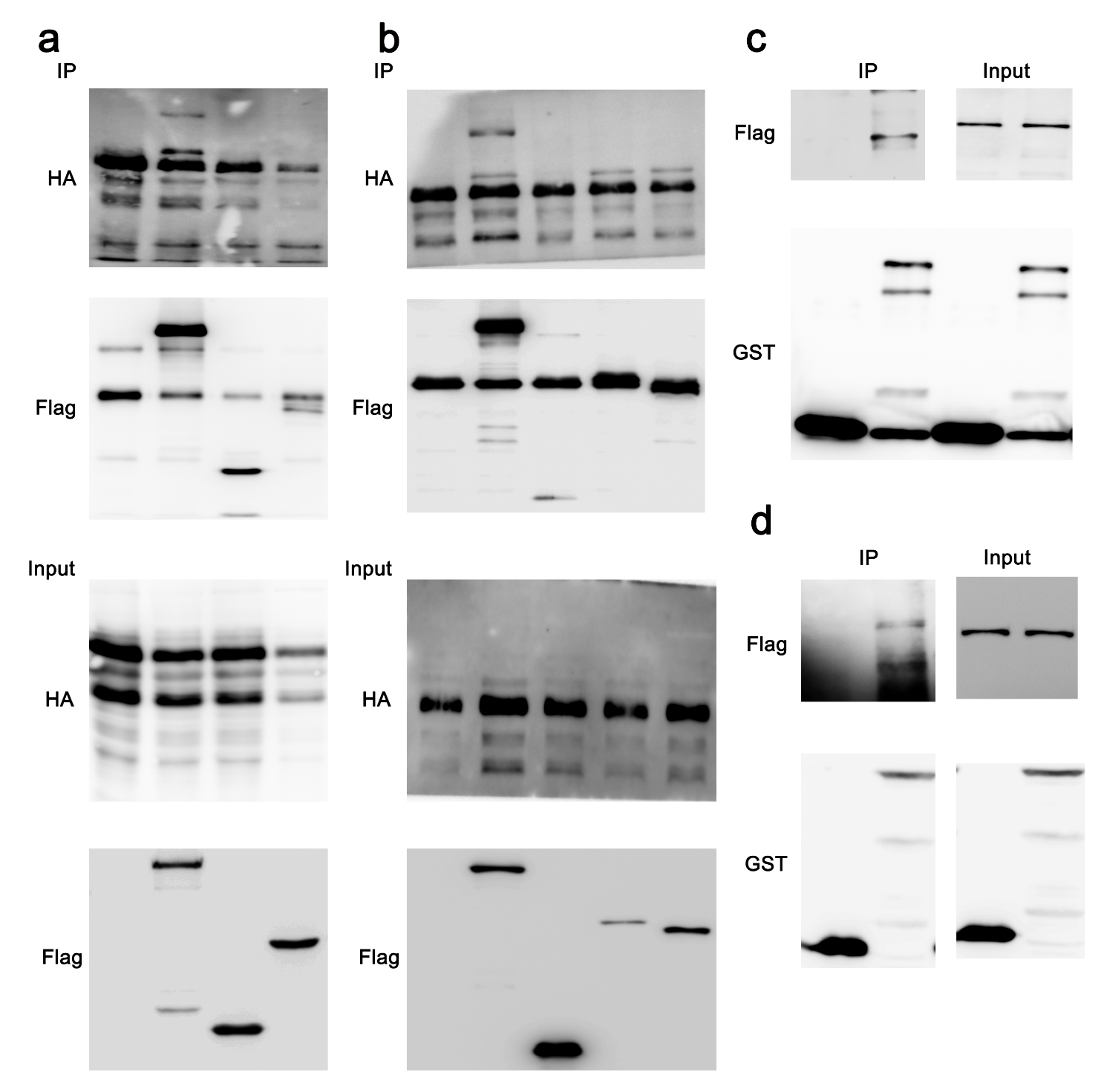


**Fig S1. Full Western blots used for Fig.1b, c, d, e.**

(a) Full Western blots used for Fig.1b. (b) Full Western blots used for Fig.1c. (c) Full Western blots used for Fig.1d. (d) Full Western blots used for Fig.1e.


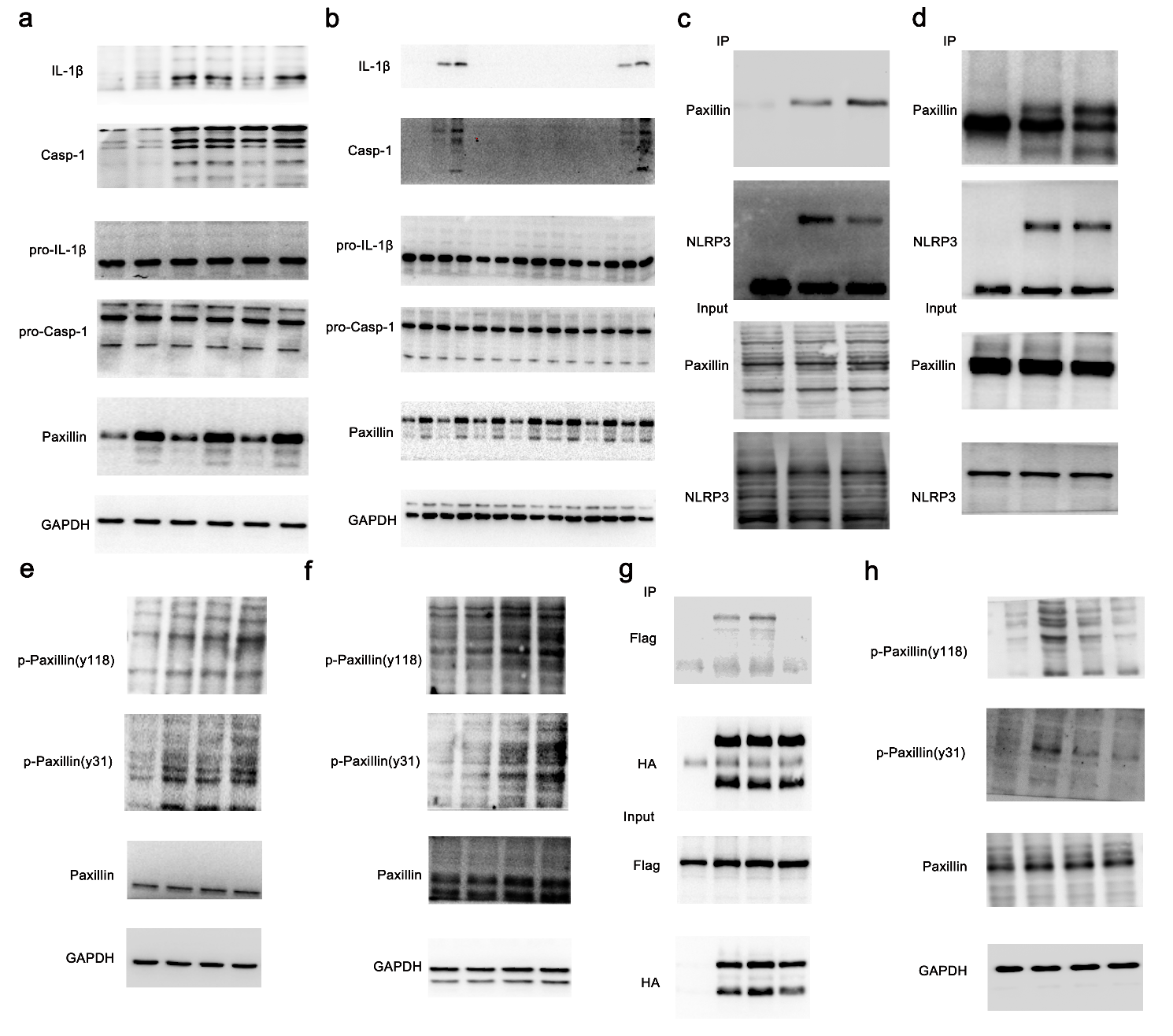


**Fig S2. Full Western blots used for Fig.2b, d, e, f, g, h, i, j.**

(a) Full Western blots used for Fig.2b. (b) Full Western blots used for Fig.2d. (c) Full Western blots used for Fig.2e. (d) Full Western blots used for Fig.2f. (e) Full Western blots used for Fig.2g. (f) Full Western blots used for Fig.2h. (g) Full Western blots used for Fig.2i. (h) Full Western blots used for Fig.2j.


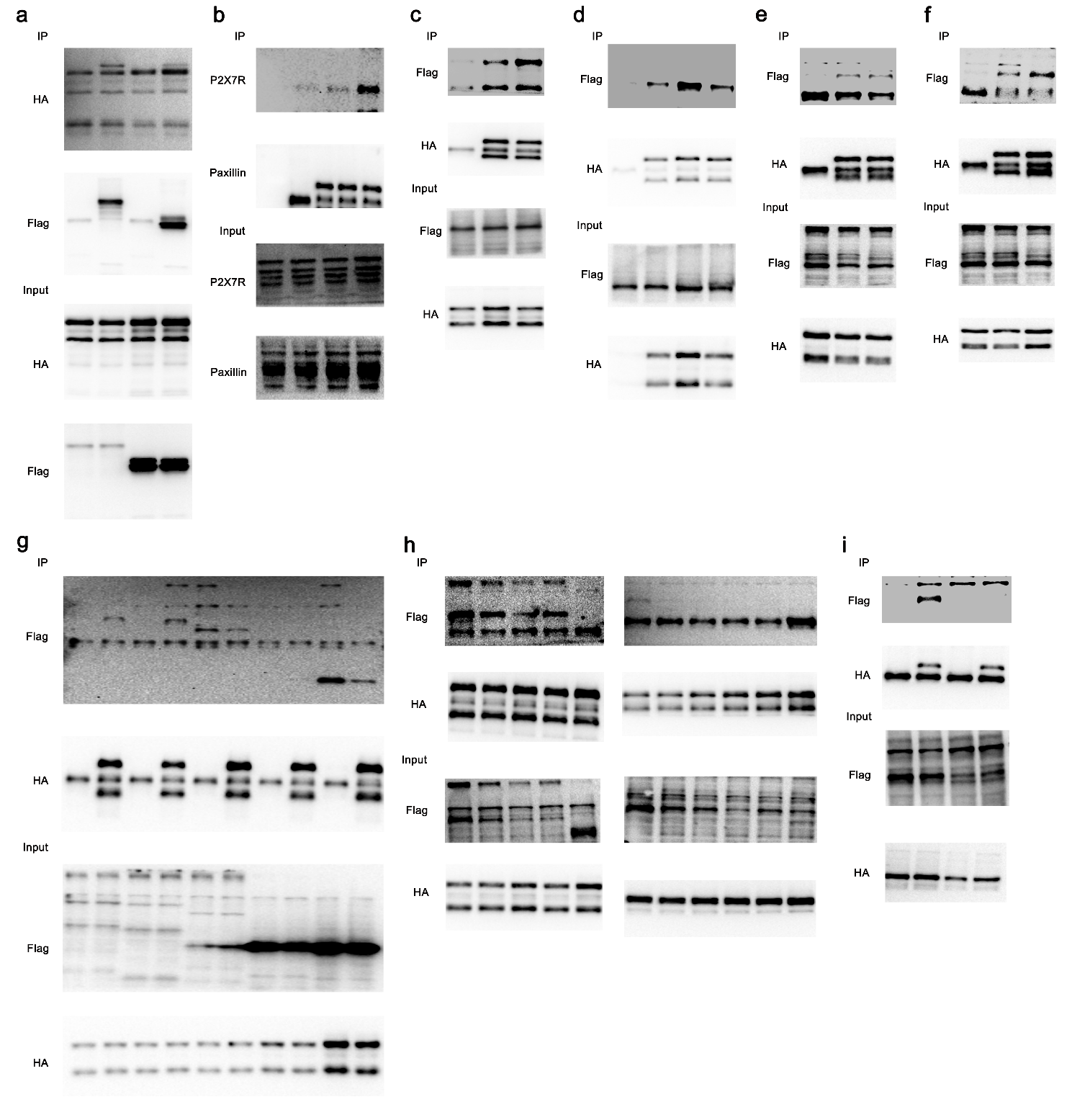


**Fig S3. Full Western blots used for Fig.3a, b, c, d, e, f, h, j, k.**

(a) Full Western blots used for Fig.3a. (b) Full Western blots used for Fig.3b. (c) Full Western blots used for Fig.3c. (d) Full Western blots used for Fig.3d. (e) Full Western blots used for Fig.3e. (f) Full Western blots used for Fig.3f. (g) Full Western blots used for Fig.3h. (h) Full Western blots used for Fig.3j. (i) Full Western blots used for Fig.3k.


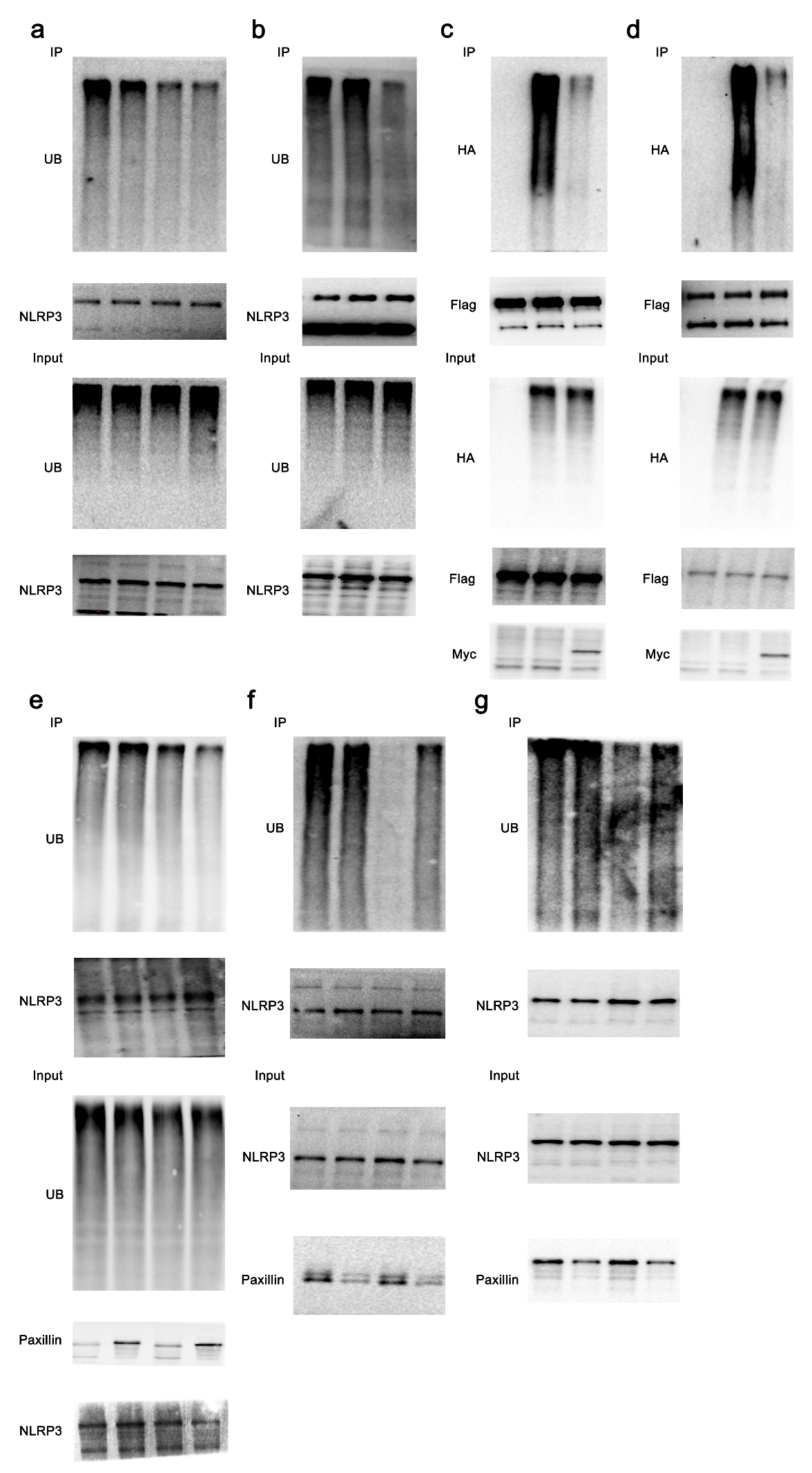


**Fig S4. Full Western blots used for Fig.4a, b, c, d, e, f, g.**

(a) Full Western blots used for Fig.4a. (b) Full Western blots used for Fig.4b. (c) Full Western blots used for Fig.4c. (d) Full Western blots used for Fig.4d. (e) Full Western blots used for Fig.4e. (f) Full Western blots used for Fig.4f. (g) Full Western blots used for Fig.4g.


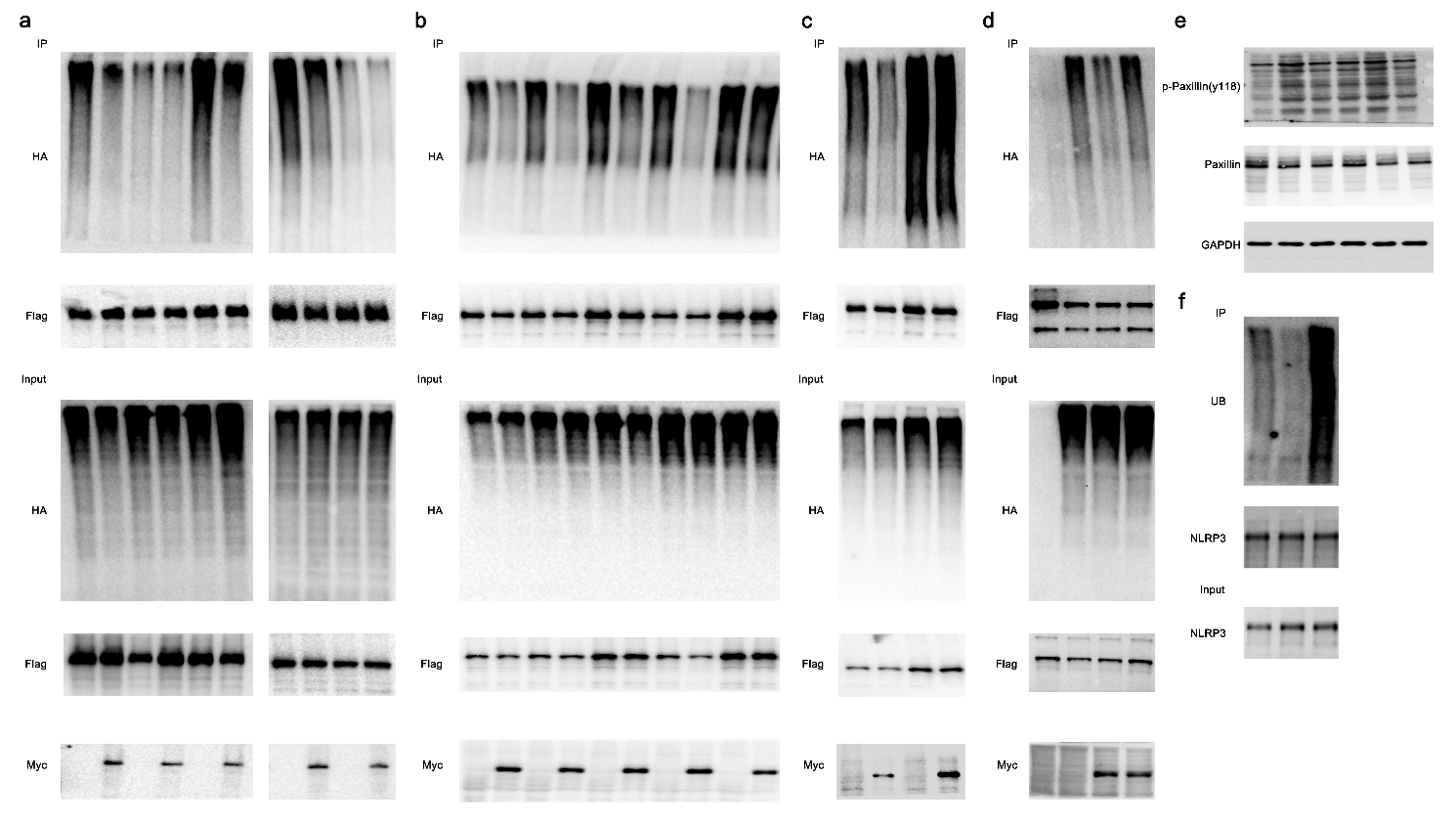


**Fig S5. Full Western blots used for Fig.5a, b, c, d, e, f.**

(a) Full Western blots used for Fig.5a. (b) Full Western blots used for Fig.5b. (c) Full Western blots used for Fig.5c. (d) Full Western blots used for Fig.5d. (e) Full Western blots used for Fig.5e. (f) Full Western blots used for Fig.5f.


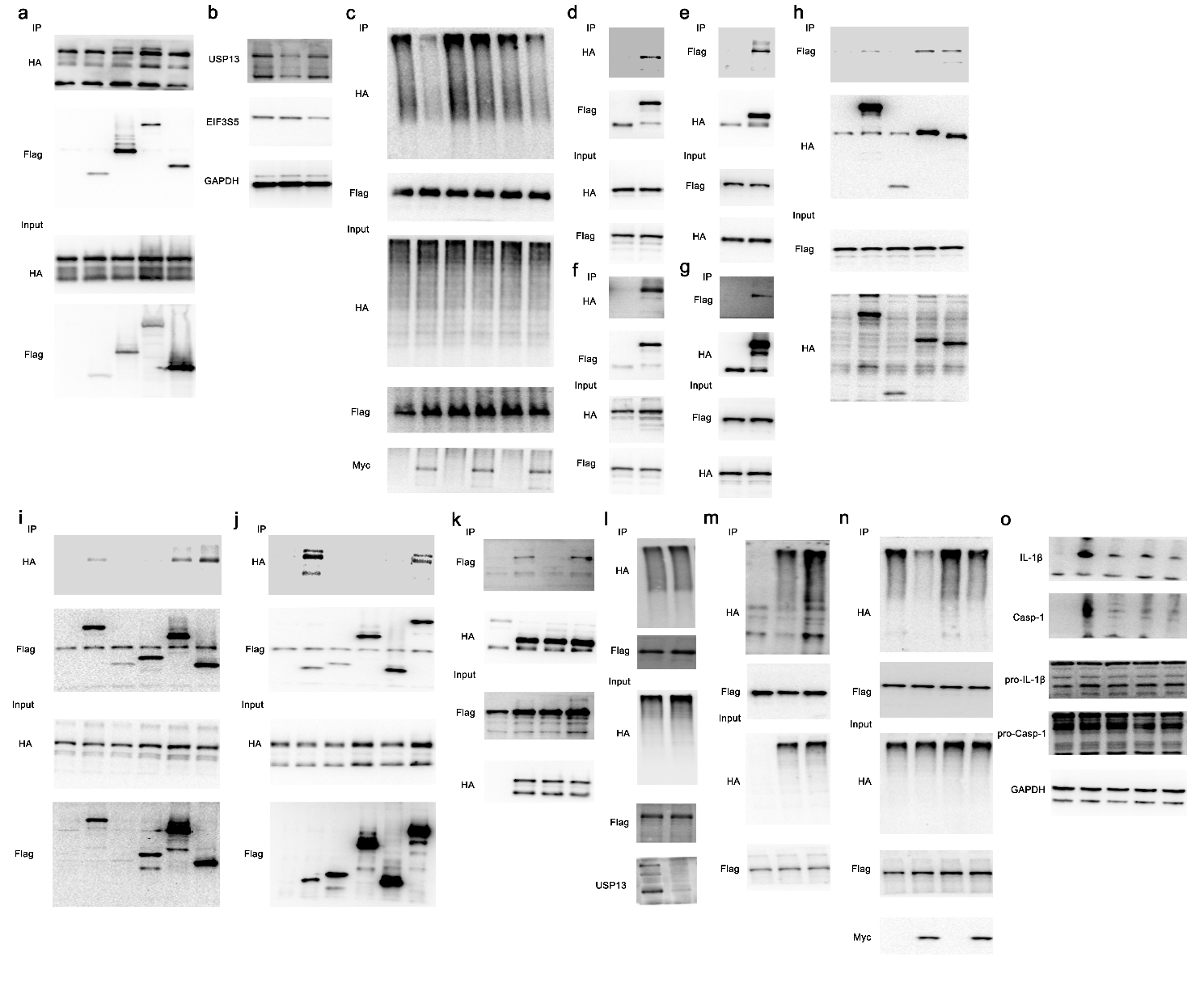


**Fig S6. Full Western blots used for Fig.6a, b, c, d, e, f, g, h, j, k, l, m, n, o, q.**

(a) Full Western blots used for Fig.6a. (b) Full Western blots used for Fig.6b. (c) Full Western blots used for Fig.6c. (d) Full Western blots used for Fig.6d. (e) Full Western blots used for Fig.6e. (f) Full Western blots used for Fig.6f. (g) Full Western blots used for Fig.6g. (h) Full Western blots used for Fig.6h. (i) Full Western blots used for Fig.6j. (j) Full Western blots used for Fig.6k. (k) Full Western blots used for Fig.6l. (l) Full Western blots used for Fig.6m. (m) Full Western blots used for Fig.6n. (n) Full Western blots used for Fig.6o. (o) Full Western blots used for Fig.6q.


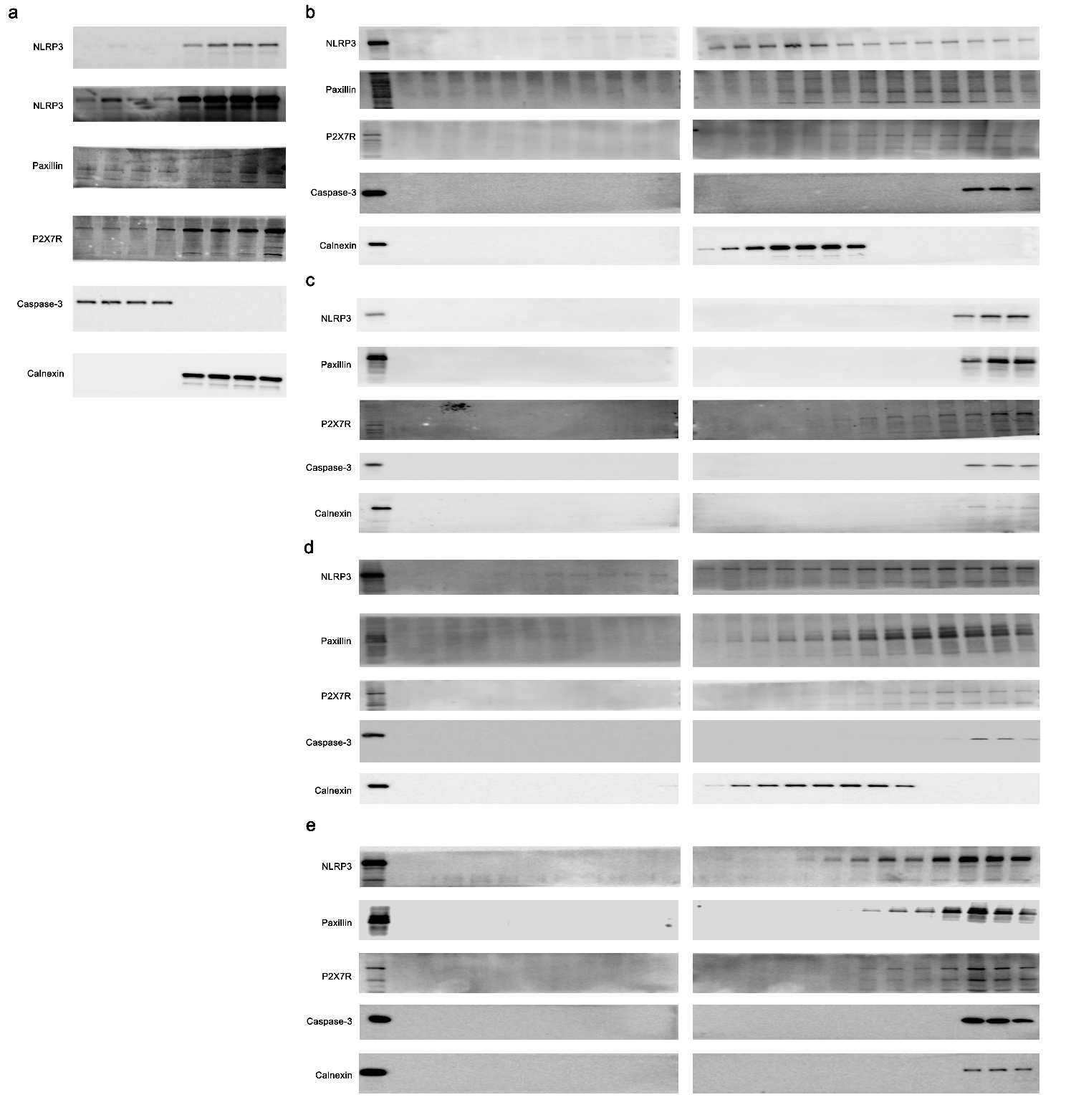


**Fig S7. Full Western blots used for Fig.8a, b, c, d, e.**

(a) Full Western blots used for Fig.8a. (b) Full Western blots used for Fig.8b. (c) Full Western blots used for Fig.8c. (d) Full Western blots used for Fig.8d. (e) Full Western blots used for Fig.8e.


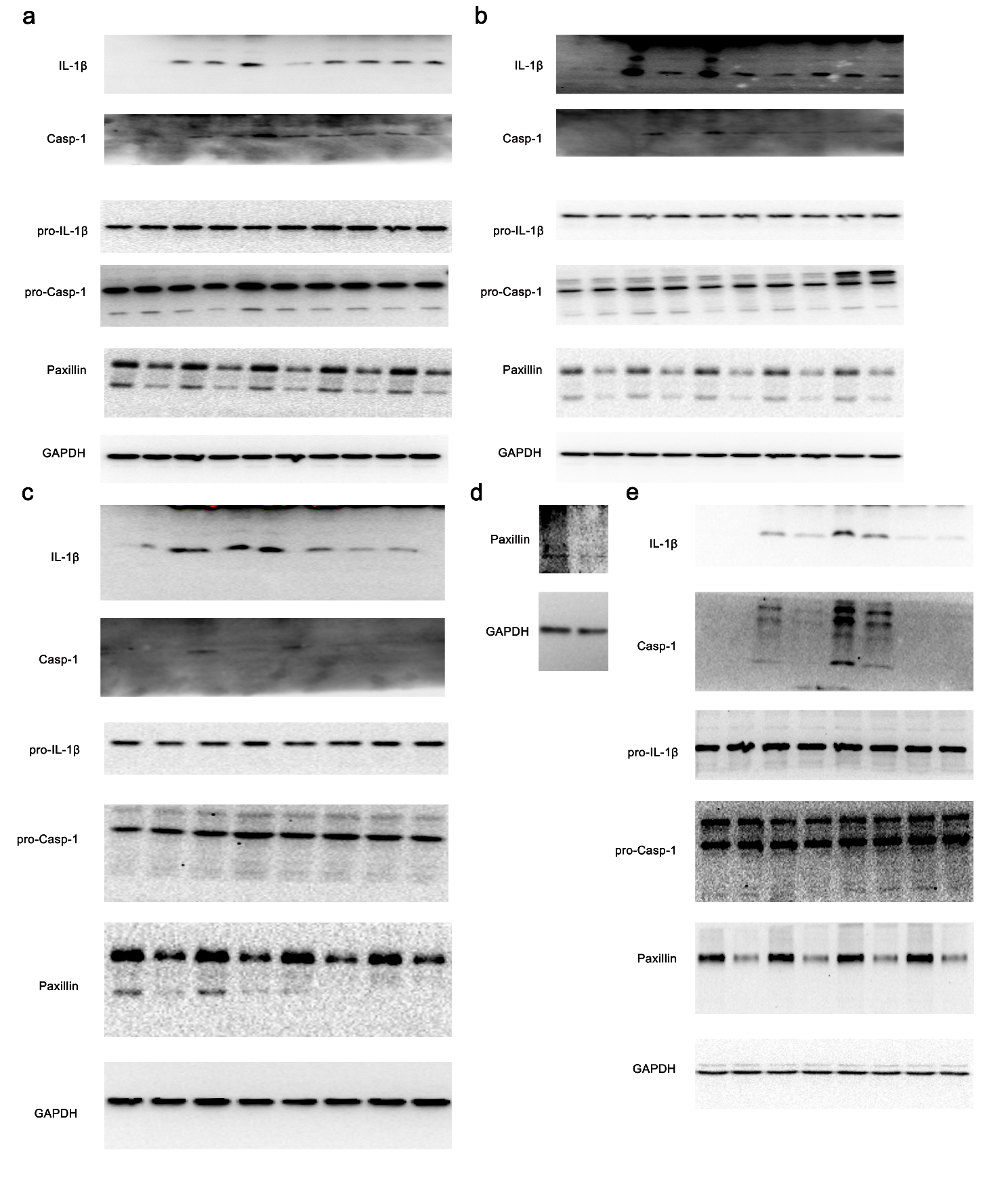


**Fig S8. Full Western blots used for Fig.9b, d, f, g, j.**

(a) Full Western blots used for Fig.9b. (b) Full Western blots used for Fig.9d. (c) Full Western blots used for Fig.9f. (d) Full Western blots used for Fig.9g. (e) Full Western blots used for Fig.9j.
